# Edible Insects as Future Proteins: Nutritional Value, Functional Properties, Bioactivities, and Safety Perspectives

**DOI:** 10.3390/nu17193165

**Published:** 2025-10-07

**Authors:** Xinyan Xu, Mengmeng Feng, Tongwei Wei, Fei Pan, Liang Zhao, Lei Zhao

**Affiliations:** 1 Key Laboratory of Geriatric Nutrition and Health, Beijing Technology and Business University, Ministry of Education, Beijing 100048, China; xuxy2023@163.com; 2 Department of Food Science and Chemical Engineering, Heze Vocational College, Heze 274000, China; 19846931889@163.com; 3 China International Engineering Consulting Corporation, Beijing 100080, China; wtw0885@126.com; 4 State Key Laboratory of Resource Insects, Institute of Apicultural Research, Chinese Academy of Agricultural Sciences, Beijing 100093, China; yunitcon@yeah.net

**Keywords:** edible insects protein, sustainable protein source, bioactive peptides, functional properties, allergenicity, food applications

## Abstract

The growing demand for sustainable and nutritionally balanced protein sources has intensified global interest in edible insects as an emerging alternative to conventional animal- and plant-based proteins. This review synthesizes current knowledge on insect proteins with a clear focus on four dimensions: nutritional value, functional properties, bioactivities, and safety considerations. Edible insects such as *Bombyx mori, Acheta domesticus* (*A. domesticus*), *Tenebrio molitor*, and *Hermetia illucens* provide high-quality proteins rich in essential amino acids, with favorable digestibility and bioavailability. Their unique functional characteristics—including solubility, emulsification, foaming, and gelation—support versatile applications in food formulations ranging from meat analogs to protein-fortified products. Insect-derived peptides further exhibit diverse bioactivities, such as antioxidant, anti-hypertensive, antidiabetic, and antimicrobial effects, highlighting their potential as functional food ingredients. Nevertheless, allergenicity and consumer acceptance remain critical challenges that must be addressed through improved processing technologies and regulatory frameworks. By systematically integrating these perspectives, this review underscores the promise of insect proteins as future food and health resources while outlining key barriers and research priorities for their safe and sustainable utilization.

## 1. Introduction

With the global population expected to reach 10 billion by 2050, as the FAO (Food and Agriculture Organization) forecasted, the challenge of meeting the rising demand for high-quality proteins in human nutrition and animal feed is becoming increasingly urgent. Currently, the production efficiency of most commercially available food products rich in edible proteins, such as dairy, legumes, and meat, is relatively low, and these products are associated with specific issues [1]. For example, some consumers are allergic to proteins in dairy products (such as casein and whey proteins), which can trigger an immune system reaction, causing symptoms like rashes and difficulty breathing [2]. Moreover, the excessive production of livestock-derived meat proteins imposes severe environmental consequences, including water depletion, ecosystem degradation, soil contamination, and biodiversity loss [3], while also contributing to human health risks such as elevated saturated fats and cholesterol [4]. Although fish and seafood offer more favorable fatty acid profiles and relatively lower environmental impacts, their large-scale production is constrained by overfishing, habitat degradation, and sustainability concerns [5]. Similarly, despite being widely explored as alternatives, plant proteins still exhibit intrinsic limitations, including imbalanced amino acid profiles, reduced digestibility, and potential allergenicity. Notably, soybean proteins are recognized as a major food allergen, posing additional safety concerns for sensitive populations [6]. These shortcomings underscore the urgent need to explore novel protein sources that provide superior sustainability, a more comprehensive amino acid composition, and a reduced environmental footprint.

Insect proteins are rich in essential amino acids with a well-balanced composition, serving as high-quality complete dietary proteins. Nutritionally, they outperform many traditional plant proteins and even compare favorably with some animal proteins [7,8,9]. Furthermore, their production process exhibits significant environmental advantages: requiring less land and water resources, emitting fewer greenhouse gases, and promoting a circular economy model [10]. Thus, they are regarded as an up-and-coming protein source for the future.

Regarding applications, insects have a long history as alternative protein sources. In Asia, Africa, and Latin America, over 2100 insect species have been recognized as edible and utilized to alleviate periodic shortages of conventional proteins [11]. Common species such as silkworm pupae, crickets, and mealworms have been extensively studied and confirmed as high-quality protein sources. Their application scenarios are expanding from the food industry to the pharmaceutical sector, demonstrating innovative potential.

However, the large-scale application of insect proteins in the food industry still faces several challenges, including allergenic risks, instability of functional properties, and the absence of comprehensive regulatory frameworks [12]. To address these issues, this review systematically summarizes the familiar sources, nutritional values, functional properties, and bioactivities of insect proteins, evaluates their application potential, discusses allergenic risks and desensitization strategies, and examines the current status of relevant legislation. The aim is to provide a scientific reference to support their safe utilization and to promote the sustainable development of the insect protein industry.

This review primarily retrieved literature from databases including PubMed, Web of Science, and Scopus. Keywords such as “edible insects,” “insect protein,” “nutritional value,” “functional properties,” “bioactivity,” and “safety” were used. Articles published in English from 2000 to 2025 were considered. Priority was given to peer-reviewed original research and review articles that provided experimental data or comprehensive evaluations relevant to edible insects’ nutritional and functional aspects.

## 2. Nutritional Characteristics of Edible Insects and Their Protein Fractions

### 2.1. Nutritional Characteristics of Edible Insects Subsection

Edible insects have gained growing recognition as a sustainable nutritional resource, attributed to their rich species diversity, robust adaptability, and concentrated nutritional composition. Their nutritional value is shaped by various factors, such as insect species, geographical origin, developmental stage, and feeding patterns [13]. Table 1 lists the nutrients in common insects. Among the major nutrients, protein is the most abundant component in insects, with crude protein content ranging from 21% to 80% of dry weight, and most species falling within the 20% to 70% range [14]. Analyses of nearly one hundred edible insect species have demonstrated that insects exhibit high crude protein levels across various developmental stages (egg, larva, pupa, and adult). Notably, over 50% of insect protein is digestible under simulated human gastrointestinal conditions, indicating high digestibility and bioavailability, suggesting that insect protein is an effective supplement to conventional protein sources such as meat and soybeans [15]. Table 2 compares essential amino acid (EAA) contents in several common edible insects against the FAO standard protein reference. Insect-derived proteins are abundantly rich in EAAs, notably branched-chain amino acids (BCAAs), including leucine (Leu), isoleucine (Ile), and valine (Val), with concentrations generally surpassing those found in traditional animal and plant proteins such as whey and soy proteins. Furthermore, the high proportion of hydrophilic amino acids—such as glutamic acid (Glu) and aspartic acid (Asp)—contributes to improved protein solubility and hydration capabilities. Concurrently, the enrichment of hydrophobic amino acids like alanine (Ala) and phenylalanine (Phe) enhances interfacial adsorption, thereby providing a molecular foundation for favorable functional properties such as emulsification and gelation [16].

Lipids constitute the second most prevalent nutrient category in edible insects, typically comprising 10% to 50% of their dry weight. Higher lipid contents are frequently noted during the larval developmental stage. Insect-derived lipids are primarily composed of unsaturated fatty acids, including omega-3 (ω-3) and omega-6 (ω-6) fatty acids, which have been linked to potential cardiovascular health advantages [17]. Moreover, the fatty acid profiles vary among insect species; however, most species exhibit relatively high levels of several fatty acids, including palmitic acid (which, while a saturated fatty acid, is a significant energy source and a crucial precursor for metabolic synthesis [18]), alongside the recognized beneficial unsaturated fatty acids such as oleic acid, linoleic acid, and α-linolenic acid [19].

Carbohydrates exist in relatively limited quantities in insects. Nevertheless, structural polysaccharides like chitin (and its derivative chitosan) demonstrate significant physiological functions. Chitin, a polymer composed of N-acetylglucosamine units, is the insect exoskeleton’s main structural component. It is acknowledged as the second most abundant natural polysaccharide in the natural world, trailing only cellulose [20]. It has been reported to exert multiple biological functions, including immunomodulatory effects, promoting hepatocyte regeneration, enhancing gastrointestinal health, and inhibiting pathogenic microbial growth [21].

Regarding mineral composition, edible insects are rich in essential trace elements, including iron, zinc, copper, manganese, and selenium. They also contain substantial macro-elements such as calcium, magnesium, potassium, sodium, and phosphorus. Notably, the phosphorus content and its bioavailability are relatively high. For example, studies have shown that the absorption rate of phosphorus in insect larvae can reach up to 92% [19]. These minerals are vital in maintaining bone health, supporting immune function, and regulating various metabolic processes. Given the distinctive nutritional profile of edible insects, a comprehensive understanding of the characteristics of commonly consumed species is essential for evaluating their practical potential and application value in the food industry. The aforementioned nutritional characteristics vary among different insect species. The following section will focus on several common edible insects, providing a detailed analysis of the nutritional profiles and application potential of their proteins. The aforementioned nutritional characteristics vary among different insect species. The following section will focus on several common edible insects, providing a detailed analysis of the nutritional profiles and application potential of their proteins.

**Table 1:** Proximate compositions and micronutrients (minerals and vitamins) of some edible insects (based on dry matter).

		*Bombyx mori*	*Hermetia illucens*	*Acheta domesticus*	*Tenebrio molitor*	*Locusta migratoria*	*Omphisa fuscidentalis*	Formicidae
Proximate composition	Proteins (%)	48.70–58.00	41.44	64.38–70.75	47.70–49.08	55–65	42–67	29.89–39.09
	Fat (%)	30.10–35.00	35.69	18.55–22.8	35.17–37.7	10–20	11.29	55.9
	Fiber (%)	2.00	0.08	-	5.00–14.96	3–7	-	3–5
	Ash (%)	4.00–8.60	7.87	3.57–5.10	2.36–3.00	3–6	5.72	1.39
	Carbohydrates (%)	1.00	12.85	2.60	7.09–7.10	5–10	-	-
	Energy (kJ/kg)	23,236.74	-	19,057.89	22,863.14	4000–5000	3500–4500	-
Minerals (mg/100 g)	Calcium	158.00	2295.00	132.14–210.00	44.36–47.18	65.6	129.5	88
	Potassium	-	478.00	1126.62	761.54–895.01	349.8	536.5	262
	Magnesium	207.00	220.00	80.00–109.42	210.24–221.54	39.4	104.7	106
	Phosphorous	474.00	547.00	780.00–957.79	697.44–748.03	266.9	-	169
	Sodium	-	204.00	435.06	125.38–140.94	515.9	103.5	-
	Iron	26.00	27.00	6.27–11.23	5.41–5.51	3.45	-	5.7
	Zinc	23.00	6.90	18.64–21.79	11.41–13.65	8.22	14.005	10.9
	Manganese	0.71	13.06	2.97–3.73	0.92–1.36	1.07	-	4.18
	Copper	0.15	1.12	0.85–2.01	1.60–1.64	1.69	2.7	1.11
	Selenium	0.15	0.07	0.60	0.03–0.07	0.0099	0.044	-
Vitamins	Retinol (μg/100 g)	-	118.00	24.33	-	0.01–0.1	-	0.01–0.05
	α-Tocopherol (IU/kg)	-	80.39	63.96–81.00	-	1–5	0.5–2	1–3
	Ascorbic acid (mg/100 g)	-	-	9.74	3.15–6.15	1–5	1–3	1–4
	Thiamin (mg/100 g)	-	-	0.13	0.31–0.63	0.2–0.5	0.1–0.3	0.1–0.4
	Riboflavin (mg/100 g)	-	-	11.07	0.41–2.13	0.3–0.7	0.1–0.4	0.1–0.5
	Niacin (mg/100 g)	0.95	-	12.59	10.59–10.68	1–2	0.5–1.5	2.02
	Pantothenic acid (mg/100 g)	-	-	7.47	3.72–6.88	0.5–1	-	-
	Biotin (μg/100 g)	-	-	55.19	78.74–94.87	-	-	
	Folic acid (mg/100 g)	-	-	0.49	0.30–0.41	0.01–0.05	-	0.73
Reference		[11]	[22]	[11]	[11]	[23,24]	[25]	[26,27]

**Table 2:** Essential amino acid contents of edible insects as compared with the FAO standard protein.

Source	Picture	Amino Acid Ratio	Reference
*Bombyx mori*	** 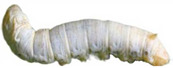 **	** 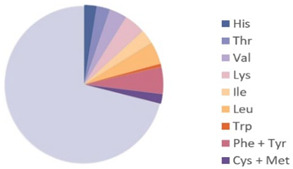 **	[9]
*Hermetia illucens*	** 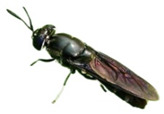 **	** 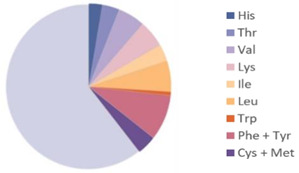 **	[28]
*Tenebrio molitor*	** 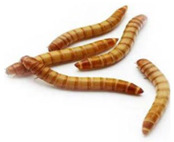 **	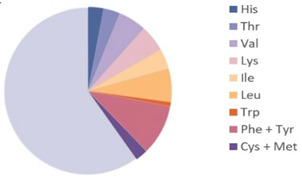	[9]
*Acheta domesticus*	** 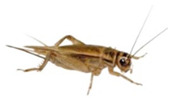 **	** 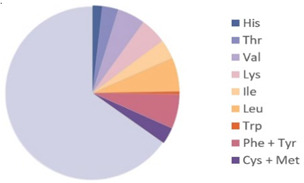 **	[9]
*Locusta migratoria*	** 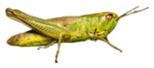 **	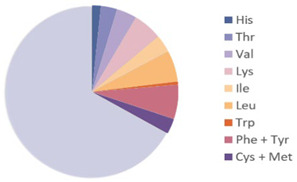	[24]
*Omphisa fuscidentalis*	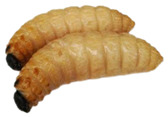	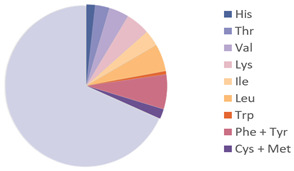	[29]
Formicidae	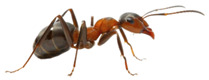	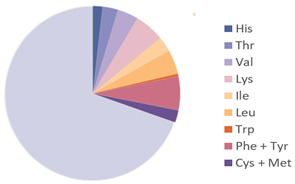	[27]
Amino Acids Required in Human Nutrition (FAO)	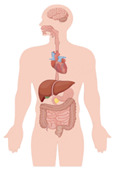	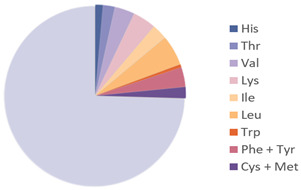	[28]

### 2.2. Common Edible Insects

#### 2.2.1. Bombyx Mori Pupae

*Bombyx mori pupae* are highly popular in many Asian countries, including China, India, Thailand, Korea, and Japan [30]. They are abundant in organic and inorganic components, including crude protein, lipids, carbohydrates, dietary fiber, vitamins, and minerals. This nutrient-dense composition positions them as a valuable food resource for alleviating nutritional deficits among undernourished populations [31,32]. The protein content of *Bombyx mori* pupae reaches approximately 55.60%, primarily derived from defatted pupal biomass. These proteins, ranging in molecular weight from 75 to 375 kDa, are mainly composed of high-quality globulins that are readily digestible and bioavailable to the human body [16]. The protein composition of *Bombyx mori* pupae is mainly derived from storage proteins, such as globulins and albumins, which account for a large proportion of the defatted pupal biomass. These proteins are highly digestible and bioavailable, making pupae a valuable nutritional resource [33]. In addition, *Bombyx mori* pupae contain distinct immune-related proteins, such as the antimicrobial peptide moricin, which features an amphipathic α-helical structure. Moricin exhibits potent antimicrobial activity against Gram-positive bacteria, especially *Staphylococcus aureus*, by disrupting bacterial membrane integrity and inducing cell death [34]. Furthermore, digestive enzymes such as trypsin are highly expressed in the midgut during larval stages and play a key role in protein digestion. Specific trypsin isoforms, such as the digestive enzyme trypsin, alkaline A (BmTA), have also demonstrated antiviral properties [35].

Proteins from *Bombyx mori* pupae are rich in 17 amino acids, including all nine EAAs required by the human body [36]. The essential to non-essential amino acid ratio stands at 0.70, closely corresponding to the optimal amino acid composition proposed by the FAO and the World Health Organization (WHO) [16]. In addition, scientific studies have shown that *Bombyx mori* pupae and eggs possess a range of bioactivities, including immunomodulatory, hepatoprotective, antitumor, anti-hypertensive, hypolipidemic, and anti-aging effects [33]. Therefore, *Bombyx mori* pupae represent a high-quality source of animal protein with significant nutritional and functional potential.

#### 2.2.2. Acheta Domesticus

The house cricket (*Acheta domesticus*) is regarded as one of the most promising edible insect species, primarily owing to its outstanding nutritional composition. This insect boasts high concentrations of protein and fat, complemented by an abundant array of vitamins and minerals [12]. *Acheta domesticus* (*A. domesticus*) has been recognized as a viable alternative protein source due to its substantial protein content. The proteins derived from crickets are composed of sufficient quantities of essential and non-essential amino acids, meeting the amino acid requirements for adults and children as defined by the WHO and the FAO [37]. The primary nutritional advantage of cricket protein lies in its high digestibility, mainly attributable to the abundance of muscle structural proteins typical of insect musculature [38]. The comprehensive functional properties of cricket protein offer significant potential for cross-disciplinary applications in the food industry, biocatalysis, and pharmaceutical development. Regarding mineral composition, crickets are rich in calcium, magnesium, and iron. Notably, they exhibit particularly high levels of copper (Cu), manganese (Mn), and zinc (Zn), with reported values in the ranges of 2.33–4.51, 4.1–12.5, and 12.8–21.8 mg/100 g of dry matter, respectively [39]. In addition, crickets contain substantial amounts of chitin and chitosan, which have been shown to inhibit pathogenic microorganisms in the gastrointestinal tract [40]. Crickets have already been widely incorporated into various food applications, as nutritional and functional additives and animal feed components, demonstrating their versatility and utility in sustainable nutrition strategies.

#### 2.2.3. *Tenebrio molitor*

*Tenebrio molitor* (*T. molitor*), belonging to the order *Coleoptera* and family *Tenebrionidae*, is recognized as a rich source of high-quality protein and has been referred to as a protein feed treasure. In the 1950s, *T. molitor* was introduced into China from the Soviet Union for use as animal feed. With the advancement of breeding technologies, *T. molitor* has demonstrated unique value in multiple applications, including the bioconversion of agricultural and household waste, plastic degradation, and the rearing of insect natural enemies. During the COVID-19 pandemic in 2020, when international trade was severely disrupted and China faced a shortage of protein-rich feed resources, *T. molitor* emerged as a promising and utilizable edible protein source with high research and application potential [41,42]. The protein content of *T. molitor* ranges from 63% to 69% on a dry weight basis, which is significantly higher than that of conventional meat sources [43]. It contains 18 amino acids, including all eight EAAs and histidine required by the human body. EAAs account for 39.14% of the total amino acid content, closely aligning with the FAO/WHO standard, which recommends that EAAs constitute approximately 40% of total amino acids in high-quality protein. The protein nutritional value of *T. molitor* is comparable to, or even surpasses, that of other commonly consumed animal proteins, making it a competitive emerging protein source to meet growing consumer demands [44].

The diversity of protein types in *T. molitor* is closely related to its physiological adaptability and versatile metabolic functions. Among these, storage proteins constitute over 50% of the total larval proteins and play a vital role during metamorphosis by being proteolytically cleaved into EAAs (e.g., leucine, lysine) to support development [45]. Apolipoproteins facilitate lipid transport and enhance the larvae’s ability to convert high-fat waste into usable energy [38]. In addition, *T. molitor* secretes various digestive enzymes, including serine proteases (TmSP1/TmSP2) and cysteine proteases, which exhibit broad substrate specificity and efficiently degrade cellulose–chitin complexes. This enzymatic capacity underpins its competitive advantage in organic waste bioconversion [46,47]. Key immune-related proteins in *T. molitor* include Tenecin 1, which shows potent activity against Gram-positive bacteria, particularly methicillin-resistant *Staphylococcus aureus* [48]. Chitinase contributes to molting and degrades fungal cell walls, offering potential for antifungal drug development [49]. Antifreeze proteins enhance cold tolerance and help regulate water balance [50]. Together, these proteins support the insect’s nutritional value and its applications in biomedical materials (e.g., chitosan dressings) and industrial biocatalysis [41].

#### 2.2.4. Hermetia Illucens

The larvae of *Hermetia illucens* (Black Soldier Fly, BSF) represent one of the most promising insect species for large-scale production [51]. *H. illucens* larvae exhibit the ability to feed on and digest diverse organic substrates, such as slaughterhouse waste, animal by-products, and brewery waste. These larvae can transform such waste materials into nutrient-dense biomass, effectively mitigating agricultural and other organic waste burdens [30]. In their prepupal or pupal stages, *H. illucens* larvae serve as a valuable source of proteins (37% to 63% on a dry matter basis) [36,52] and polyunsaturated fatty acids (7% to 39% on a dry matter basis) [53,54], among other essential nutrients. Furthermore, the amino acid composition of *H. illucens* larvae meets the requirements set by the WHO, making them an excellent alternative protein source [32].

The protein composition of *H. illucens* larvae is predominantly composed of muscle proteins (such as actin and myosin) and structural proteins (e.g., epidermal proteins, chitin-binding proteins), which account for 60–70% of total protein content. The high proportion of myofibrillar proteins imparts a texture to the larval tissue like that of animal meat. It is particularly beneficial in developing plant-based meat alternatives, where it can substitute soy protein isolate [55]. The storage proteins of *H. illucens* are rich in sulfur-containing amino acids (methionine, cysteine), which complement the amino acid profiles of plant proteins, making them an ideal ingredient for infant formula [51]. Additionally, the functional enzymes of *H. illucens* larvae, such as chitinase and lipase, participate in the rapid degradation of high-fat waste and retain activity during processing, serving as feed additives to enhance the digestibility of livestock and poultry [56]. Furthermore, the antimicrobial peptides of *H. illucens* (e.g., HC1 and HC10) can bind to lipopolysaccharides (LPS) from Gram-negative bacteria, reducing LPS-induced inflammatory responses. This demonstrates their potential in combating Gram-negative bacterial infections [57]. In sustainable food systems, *H. illucens* protein, owing to its high gelation, emulsifying properties, and low allergenicity, has been successfully applied in plant-based meat binders and protein beverage stabilizers [7,58,59]. Identifying numerous structural and muscle proteins in *H. illucens* larvae further underscores the potential of their protein as a substitute for meat and soy protein in various applications [55]. Therefore, products derived from *H. illucens* larvae hold considerable promise as alternatives to traditional food sources and are poised to become a significant protein-rich dietary substitute in the future.

#### 2.2.5. Locusta Migratoria

*Locusta migratoria* (*L. migratoria*), a long-standing pest in global agricultural ecosystems, has recently garnered attention as a sustainable protein resource due to its unique nutritional and ecological value [24]. Studies have shown that dried *L. migratoria* contains over 62% protein, making it a rich source of high-quality protein [60]. The protein in *L. migratoria* comprises 17 amino acids, classifying it as a complete protein. Notably, EAAs account for over 30% of the total amino acid content, with well-balanced proportions of limiting amino acids such as lysine and threonine, and a bioavailability superior to most plant proteins [61]. The protein profile of *L. migratoria* is particularly distinguished by the synergistic functions of resilin and the lipid transport protein ApoLp-I/II. Resilin, an elastic protein found in the wing hinge of locusts, imparts rubber-like elasticity, enabling highly efficient jumping and wing articulation. It is considered one of the most efficient elastic proteins, with an energy return efficiency of up to 97% [39]. ApoLp-I/II, a major apolipoprotein in insects, is involved in the transport and deposition of cuticular lipids, playing a critical role in maintaining the integrity of the insect cuticle barrier [62]. In addition, locust storage proteins, including Hexamerin-90 and Hexamerin-270, exhibit distinct hormone-regulated expression patterns during development and are especially important during ovarian maturation and vitellogenesis [63].

Moreover, over 85% of locust fat consists of soft and unsaturated fatty acids, with a digestibility rate exceeding 90%, low fiber content (<3%), and high levels of micronutrients such as iron and zinc, offering both high nutritional density and metabolic efficiency [61]. Locust-derived compounds have demonstrated hypolipidemic, anti-fatigue, antioxidant, immunomodulatory, and anti-aging effects. Furthermore, insect proteins and peptides have exhibited anti-inflammatory and antioxidant activities, making them promising functional ingredients for developing health-promoting foods [31]. Of particular interest is the emerging “pest valorization” strategy, which proposes that large-scale rearing of locusts can reduce their impact in agricultural fields through controlled farming and decrease reliance on chemical pesticides, thereby indirectly preserving agroecosystem health [64]. This paradigm shift repositions locusts from being solely agricultural pests to dual-purpose resources—serving both as a high-quality protein source and a tool for agroecological regulation—offering innovative solutions for global protein shortages and sustainable agricultural development.

#### 2.2.6. Formicidae

Formicidae, belonging to the class Insecta, order Hymenoptera, are a dominant group of terrestrial organisms and represent a typical example of eusocial insects. Globally, more than 15,000 Formicidae species have been identified. According to the classification system proposed by Bolton (2003), Formicidae comprises 21 extant subfamilies and four fossil subfamilies [65]. Over ten Formicidae species have been recognized for their edible and medicinal potential, while fewer than twenty are currently applied in clinical contexts. Formicidae are among the most abundant insects in nature, with studies showing that their crude protein content exceeds 50% [66]. Formicidae proteins contain 18 free amino acids, including all eight EAAs required by the human body, namely isoleucine, leucine, lysine, methionine, phenylalanine, tryptophan, threonine, and valine, which must be obtained exogenously through diet [67]. Additionally, Formicidae are rich in trace elements essential to human health, with at least 14 identified. Zinc is particularly abundant, with a content ranging from 120 to 198 mg per kilogram of dried Formicidae biomass. Formicidae contain formic acid, a bioactive compound demonstrating potential anti-rheumatic and antimicrobial activities in preliminary studies. However, it should be noted that formic acid can be toxic at high concentrations, and its safety for medicinal applications remains to be rigorously evaluated [68,69,70].

The protein profile of Formicidae is characterized by venom proteins and functionally specialized proteins involved in social behavior. In worker Formicidae, venom glands secrete formicase, an enzyme that synthesizes concentrated formic acid. This biosynthesis process primarily utilizes amino acids such as serine and glycine as precursors, conferring antimicrobial and defensive functions [71]. Social behavior in Formicidae relies on pheromone-binding proteins, particularly odorant-binding proteins (OBPs), which exhibit high affinity and stability, making them ideal candidates for highly sensitive volatile organic compound detection platforms [72]. Furthermore, Formicidae possess three significant categories of storage proteins: hexamerins, glutamine/glutamate-rich proteins, and very high-density lipoproteins. These proteins are also present in the fat bodies during queen nuptial flights, suggesting their critical roles in Formicidae development and nest establishment [73]. Formicidae’s multifunctional protein network spans biomedical, environmental, and agricultural domains. In particular, the targeted modification of venom and pheromone-related proteins presents promising opportunities for precision applications in biotechnology.

#### 2.2.7. Omphisa Fuscidentalis

*Omphisa fuscidentalis* Hampson, an insect belonging to the phylum Arthropoda, class Insecta, order Lepidoptera, family Crambidae, and genus Omphisa, is primarily distributed across Southeast Asian countries such as Myanmar, Thailand, and Laos. This species, commonly known as the bamboo caterpillar, is rich in high-quality protein, with protein content typically ranging from 25% to 35% of its dry weight, depending on environmental and rearing conditions [74,75]. The protein fraction constitutes a significant proportion of EAAs, including lysine, leucine, and isoleucine—critical nutrients for human health that must be acquired through dietary sources [29]. In addition to its protein profile, *O. fuscidentalis* exhibits a relatively high lipid content, typically ranging from 25% to 30% of its dry weight. Most of these lipids consist of unsaturated fatty acids, such as omega-3 and omega-6, which are recognized for their beneficial impacts in reducing cardiovascular disease risk. Furthermore, this insect species is rich in monounsaturated fatty acids (MUFAs) and polyunsaturated fatty acids (PUFAs), playing pivotal roles in regulating lipid homeostasis within the human body [74,76]

In *O. fuscidentalis*, two key storage proteins—OfSP1 (75 kDa) and OfSP2 (72 kDa)—have been characterized. These proteins are predominantly synthesized in the fat bodies of fourth- and fifth-instar larvae, with accumulation observed in the hemolymph during the initial stage of larval diapause [77]. Additionally, two immune-related proteins, OfAMP1 and OfAMP2, play a critical role in the insect’s defense system. These antimicrobial peptides are involved in immune responses against fungal infections and exhibit inhibitory activity against various fungal pathogens, including phytopathogenic and human pathogenic fungi.

## 3. Functional Properties of Insect Proteins

Insect proteins offer not only high nutritional value but also promising functional properties for food applications. Key attributes such as solubility, emulsification, foaming, and gelation play important roles in determining their performance in food systems (Table 3).

### 3.1. Solubility

Solubility is a key functional attribute of insect protein for food processing and product development, as it directly affects its dispersibility, stability, and ingredient compatibility during manufacturing. These factors ultimately define the end product’s texture, palatability, and nutrient preservation while creating opportunities for diverse applications in the food industry, including beverages, seasonings, and health supplements [78]. Thongkaew et al. researched protein extraction from four edible insects (*A. domesticus*, *Gryllus bimaculatus*, *Holotrichea* sp., and *Gryllotalpa orientalis*), evaluating their chemical and functional properties. They found that insect protein concentrate (IPC) exhibits high solubility. Incorporating 2–8% IPC into rice noodles can enhance their tensile strength (with the 8% group reaching a maximum of 0.17 N), improve texture, and mouthfeel. Additionally, the high protein content (73–77%) of IPC and its rich essential amino acids (such as leucine and lysine) significantly elevate the nutritional value of rice noodle products [79]. Increasing research has revealed that several factors, including temperature, ionic strength, protein concentration, oxidation–reduction status, lipid content, and moisture content, play a significant role in influencing the solubility of insect proteins. These factors affect protein conformation and interactions, affecting their solubility and stability [80]. The impact of temperature on protein solubility is complex. Generally, increased temperature enhances protein solubility by promoting molecular motion, but excessively high temperatures can cause protein denaturation, leading to insoluble aggregates and reduced solubility [81]. Hall et al. studied the functional properties of tropical banded cricket (*Gryllodes sigillatus*) protein hydrolysates. They found that mild heating improved insect protein hydrolysates’ solubility, but solubility began to decrease beyond a certain temperature threshold [82]. Ionic strength primarily affects protein solubility by influencing electrostatic interactions between protein molecules. High ionic strength can weaken electrostatic repulsion between protein molecules, increasing aggregation and reducing solubility. Yi et al. found that lower salt concentrations can enhance the solubility of yellow mealworm protein by stabilizing charged groups on the protein surface, facilitating interaction with solvents. In contrast, higher salt concentrations reduce protein solubility by competing with protein molecules for water, leading to aggregation and precipitation [83].

Employing appropriate extraction techniques can also enhance the solubility of specific edible insect proteins. Yi et al. extracted and characterized protein fractions from five insect species: *T. molitor*, *Zophobas morio*, *Alphitobius diaperinus*, *A. domesticus*, and *Blaptica dubia*. The research revealed that solubility could be improved via a simple water-based extraction protocol [84]. Kim et al. found that specific extraction methods, such as saltwater solutions, can significantly improve the solubility of proteins derived from edible insects (*T. molitor*, *Allomyrina dichotoma*, and *Protaetia*) [85]. Kim et al. further investigated the impact of defatting methods on the physicochemical properties of proteins extracted from *H. illucens* larvae and revealed that the solubility of proteins defatted with organic solvents was superior to that of other treatments (e.g., cold pressure-defatted and water extracted) [86].

### 3.2. Water- and Oil-Holding Capacities

The water-holding and oil-holding abilities of insect proteins are key technological functional properties that significantly impact the texture and sensory attributes of food [87]. These properties demonstrate the ability of proteins to interact with water and lipids, which is crucial for consumer acceptance and preference. The insect proteins’ water-holding capacity (WHC) measures their ability to absorb and retain water under certain conditions, without releasing it under pressure [88]. This property is vital for various food applications, particularly in meat products, bakery goods, and other moisture-rich foods, where it contributes to juiciness, tenderness, and overall mouthfeel. Han et al.’s research revealed that cricket flour exhibits good WHC. Under the condition of pH 6.8, the WHC of cricket flour increases significantly with the increase in NaCl concentration (0–2.5 M), rising from approximately 2 g/g (no salt) to about 3.5 g/g (2.5 M NaCl). In laboratory tests, when added to pork sausages at a proportion of 5%, it can enhance the water retention of the product, which is likely to improve the juiciness and texture of the final product [89]. Similarly, Zielińska et al. found that the WHC of cricket and mealworm protein played a crucial role in enhancing product yield and sensory quality in food formulations [90]. Oil-holding capacity (OHC) is another key feature in enhancing taste, maintaining flavor, or improving palatability. It is essential in many food applications, such as bakery products, flour milling formulations, and meat substitutes [91,92]. OHC refers to the ability of proteins to bind with fats, and this functional property is highly correlated with their emulsification properties [93]. It is suggested that the studied insects could be a valuable resource for the food industry due to their high OHC [94]. The high OHC of insect proteins can also contribute to the calorie density of food products, making them suitable for energy-dense formulations. Several factors can affect the WHC and OHC of insect proteins, including the insect species, protein denaturation degree, pH, temperature, and ionic strength. For instance, protein denaturation often results in unfolding protein molecules and exposing hydrophobic and hydrophilic sites. These sites can interact with water and oil molecules, enhancing OHC [95]. Research by Hall et al. on black soldier fly larvae protein demonstrated that heat treatment can improve WHC by altering the protein conformation, providing more binding sites for water molecules [82]. Additionally, adjusting the pH value can change the net charge of protein molecules, thereby affecting their interactions with water and oil molecules. A study by Ndiritu et al. investigated the functionality of cricket protein in food matrices and found that pH adjustments can affect the WHC of cricket protein [96].

### 3.3. Emulsifying Properties

Emulsifying properties of proteins are generally characterized by three related but distinct parameters: emulsifying activity (EA), emulsifying capacity (EC), and emulsifying stability (ES). EA describes the initial ability of proteins to adsorb at the oil–water interface and form emulsions, reflecting the rapidity and efficiency of emulsion formation. EC refers to the maximum amount of oil that a given quantity of protein can emulsify before phase separation occurs, representing the emulsification limit. ES evaluates the ability of the formed emulsion to resist droplet coalescence and phase separation over time, reflecting emulsion stability [97]. Together, these parameters provide a comprehensive picture of protein emulsification performance. The proportion of hydrophilic and hydrophobic amino acids and the secondary structure are key determinants of these properties for insect proteins. Their unique amino acid composition and structural characteristics endow them with good emulsifying performance. The surface activity of these proteins allows them to effectively adsorb at liquid interfaces, thereby playing a crucial role in both the formation (EA and EC) and maintenance (ES) of emulsions [7]. Consequently, insect proteins can be used as emulsifiers and surfactants in many natural and processed foods, such as meat analogs, protein beverages, bakery fillings, and soy milk [98]. Research by Yi et al. on T. molitor larval protein revealed its efficacy in stabilizing water-in-oil emulsions, highlighting its suitability for seasonings and meat products [84]. Gould et al. also found that the stable emulsion formed by T. molitor protein exhibits tolerance to changes in pH, salt concentration, and temperature, with its microstructure remaining unchanged under different processing conditions. This stability indicates that yellow mealworm protein can be an efficient emulsifier in various food products [98]. Furthermore, factors such as protein concentration, pH, ionic strength, and processing conditions can be adjusted to optimize the emulsifying performance of insect proteins for specific food applications. Stone et al. investigated the effects of different processing conditions (e.g., heat treatment and pH adjustment) on the emulsifying properties of insect proteins (cricket and yellow mealworm). The results showed that treatments such as high temperature or extreme pH may cause protein denaturation or alter surface activity, negatively impacting insect proteins’ emulsifying properties [99].

### 3.4. Foaming Properties

The foaming properties of insect proteins have become an increasingly important topic in food science, driven by the quest for sustainable protein alternatives. Foaming capacity (FC) refers to the ability of a substance to generate bubbles when in contact with a liquid or gas and maintain the stability of these bubbles. This characteristic is fundamental in developing foods such as mousses, whipped ingredients, and certain baked goods, where the incorporation and stabilization of air are crucial for the desired texture and mouthfeel [93]. FC and foaming stability (FS) are the two primary considerations when evaluating the foaming properties of proteins. FC refers to the foam volume produced by a protein solution, while FS pertains to how long the foam can maintain its structure without collapsing. These properties are influenced by the protein’s ability to adsorb at the air–water interface rapidly and form a cohesive and elastic film around air bubbles [100]. Therefore, the foaming properties of proteins depend on various factors, including the protein’s surface hydrophobicity, the count of hydrophilic residues, the structural conformation (whether it is globular or non-globular), and the amino acid composition, as well as external conditions (e.g., ionic strength, temperature, and specific processing technique employed) [101]. Santiago et al. examined the foaming properties of black cricket protein isolate (BCPI) under different treatments. They found that FC increased with temperature, reaching 1070% (calculated as [(V − V_0_)/V_0_] × 100, where V_0_ is the initial liquid volume and V is the total foam volume after aeration) at 95 °C, a significant increase from the untreated BCPI (190%). FS ranged between 50% and 70%, with the 0.5M NaCl and 95 °C heat-treated group demonstrating a 35% residual foam after 30 min (compared to whey protein isolate). The enhanced FC with NaCl addition is attributed to the protein’s ability to form more elastic and cohesive films within the bubble [102]. Andrea et al. discovered variations in foaming properties among insect species, even when subjected to identical treatments. Notably, the roasted and ground mealworm powder was found to be non-foaming, while the cricket powder exhibited superior FS (86%) than faba beans (62%) and yellow pea (49%) [103]. Storage of mealworm larvae at −20 °C was shown to improve their FC and FS, likely due to the presence of antifreeze proteins that facilitated protein denaturation and ongoing hydrolysis by natural enzymes in the unfrozen residual water [99]. The study indicates that the protein surface activity influences insect protein foaming properties, which depend on their hydrophilicity/hydrophobicity, structural flexibility, and interfacial migration rates [104,105]. Research on the foaming properties of insect proteins has provided valuable insights for developing high-performance food additives and ingredients. For example, it has been reported that bread made from flour supplemented with 10% cricket powder shows the closest resemblance to wheat flour-containing bread in volume and hardness, making this an acceptable substitution ratio. Additionally, studies have tested the incorporation of mealworm powder into ice cream, which significantly enhanced the melting stability (with the melting time being twice as long as that of the non-supplemented group) and antioxidant capacity (DPPH assay, IC_50_ = 0.20 ± 0.05, representing a 5-fold improvement compared to the non-supplemented group). Moreover, the viscosity increased, resulting in a more compact structure [106,107]. These studies have also highlighted the notable variability in the FC and FS of insect proteins when subjected to different processing conditions.

### 3.5. Gelation Properties

Gelation refers to the process by which proteins aggregate and cross-link to form a three-dimensional network that traps water, creating a gel-like structure. It measures the protein’s ability to aggregate and form gels [84]. Insect proteins exhibit gelation properties significantly different from traditional animal and plant proteins. Most research on insect protein gelation has focused on developing heat-induced gels [78]. The ability of these proteins to form gels is influenced by factors such as protein concentration, temperature, pH, and ionic strength. Scholliers et al. discovered that heating temperatures and the ratio of insect to pork proteins significantly influenced gelation, with insect proteins showing a unique increase in gelation during cooling, which was not reported in previous studies or when using pork proteins alone [108]. Kim et al. found that pH variations can significantly improve the gel properties of insect-protein mixtures, making them suitable for various food applications [109]. Kumar et al. investigated the impact of ultrasound treatment on the gelation properties of black soldier fly larval proteins, revealing that ultrasonication can significantly affect the functional properties of insect proteins [110]. In developing novel food products, insect proteins with good gel-forming capabilities are crucial in substituting proteins used in traditional food formulations. However, evidence linking insect protein gelation to improved sensory perception remains conditional and highly dependent on processing parameters. For instance, the incorporation of silkworm pupae powder (5–10%) into meat analogues via a freeze alignment technique significantly enhanced hardness, chewiness, and firmness, while also promoting a fiber-like structure; yet higher addition levels led to decreased water-holding capacity and thiol content, indicating trade-offs in functionality [111]. Similarly, salt-soluble proteins extracted from Protaetia brevitarsis and T. molitor larvae improved gel strength when combined with pork myofibrillar proteins at low inclusion levels (1–2%), but higher levels resulted in reduced viscosity and limited improvements in other functional properties [112]. In addition, Alphitobius diaperinus protein powder incorporated into soy-based burger analogues achieved the highest consumer acceptance at a 5% inclusion rate. In contrast, higher levels caused undesirable color darkening and reduced acceptance [113]. These findings collectively suggest that while insect protein gelation can enhance textural and sensory qualities, such benefits are not universal and depend on narrowly defined processing conditions.

**Table 3:** Summary of the effects of different factors on insect functional properties.

Functional Characteristics	Influencing Factors	Source	Influencing Effect	Reference
Solubility	pH	*Gryllodes sigillatus*	The solubility exceeded 30% under acidic (pH 3) and neutral (pH 7) conditions, increasing to 50–90% in alkaline environments (pH > 7).	[82]
Solubility	Ionic Strength	*Tenebrio molitor*	Upon the addition of 0.1 M NaCl, the protein solubility reached 100%. However, at 1 M NaCl, the protein solubility decreased to 94.7%, although it remained higher than that under salt-free conditions (62.1%).	[83]
Solubility	pH	*Tenebrio molitor*	Protein solubility is lowest at pH 4–6 (29.6%) and reaches its highest at pH 11 (68.6%).	[83]
Solubility	Ultrasound frequency	*Bombyx mori*	Under the treatment at 40 kHz, the solubility of insect protein reached 30%, compared to 19.12% in the control group. The best solubility was achieved under the triple-frequency treatment (22/28/40 kHz), reaching 41.16%.	[114]
Solubility	Temperature	*Bombyx mori*	Under high-temperature conditions (50 °C) combined with triple-frequency ultrasound (22/28/40 kHz), the protein solubility reached 41.16%, representing a 115.27% increase compared to that at 20 °C (19.12%).	[114]
Solubility	Extraction technique	*Hermetia illucens*	The solubility was highest with the organic solvent extraction method (5.24%), followed by the cold-press defatting method (4.42%), while the water extraction method had the lowest solubility (4.16%).	[86]
Solubility	Amino acid composition	*Tenebrio molitor* *Protaetia brevitarsis*	The high proportion of hydrophobic amino acids in *Tenebrio molitor* protein (valine: 1.13%) reduces its solubility. In contrast, the salt-soluble protein of *Protaetia brevitarsis* is rich in lysine (2.35%) and glutamic acid (6.47%), enhancing hydrophilic interactions and improving solubility.	[85]
Solubility	Treatment method	*Tenebrio molitor*	Cold atmospheric plasma induced more substantial alterations in protein composition, leading to a reduction in protein solubility.	[94]
Emulsion	Extraction technique	*Protaetia brevitarsis*	The emulsifying capacity of protein extracted by the salt-soluble method (88.5%) was significantly higher than that obtained by the water-soluble method (75.2%).	[85]
Emulsion	Extraction technique	*Hermetia illucens*	The protein extracted by the cold-press method exhibited the highest emulsifying capacity (85%), followed by the organic solvent extraction method (76%), while the water-extracted protein showed the lowest emulsifying capacity (60%).	[86]
Emulsion	Temperature	*Tenebrio molitor*	Temperature was 55 °C, the EA was 28.94%, and the ES was 64.97%. Temperature was 75 °C, the EA was 37.87%, and the ES was 78.03%. Temperature was 95 °C, the EA was 38.00%, and the ES was 65.16%.	[115]
Emulsion	Temperature	*Sphenarium purpurascens Charpentier*	Temperature was 60 °C, and the EC was 20.33%. Temperature was 90 °C, and the EC was 18.5%.	[114]
Emulsion	pH	*Acheta domesticus*	At pH 6, the EC and ES were the lowest. At pH 12, the EC was 38.58%, and the ES was 33.33%.	[96]
Foaming	pH	*Blaptica dubia*	Under pH conditions of 3, 5, 7, and 10, foam formation occurred only at pH 5, with a half-life of 5 min, indicating that neutral to slightly acidic conditions are most favorable for foaming.	[84]
Foaming	Temperature	*Tenebrio molitor*	Temperature was 55 °C, the FC was 6.50%, and the FS was 94.30%. Temperature was 75 °C, the FC was 8.19%, and the FS was 93.82%. Temperature was 95 °C, the FC was 3.58%, and the FS was 97.37%.	[115]
Foaming	pH	*Acheta domesticus*	At pH 4, the FC was 14.05%, and the FS was 5.11%. At pH 6, the FC was 9.10%, and the FS was 6.01%.	[96]
Foaming	Ionic Strength	*Gryllus assimilis*	When the concentration of NaCl is 0.3 M, the foaming property is 1150%, and the FS is 25%. When the concentration of NaCl is 0.5 M, the foaming property is 1170% and the FS is 35%.	[102]
Foaming	Temperature	*Gryllus assimilis*	The foaming property of the untreated protein is 190%. The foaming property of the protein heat-treated at 75 °C is 970%, and that of the protein heat-treated at 95 °C is 1070%.	[102]
Foaming	pH	*Hermetia illucens*	At pH 6, the FC was 34.37%, and the FS was 23.81%. At pH 10, the FC was 5.26%, and the FS was 10%.	[116]
Gelation	Protein concentration	*Zophobas morio* *Blaptica dubia*	At a protein supernatant concentration of 3%, none of the insect proteins formed a gel, whereas at a concentration of 30%, all insect proteins formed a gel.	[84]
Gelation	pH	*Acheta domesticus*	At a protein concentration of 3% *w*/*v*, only the supernatant of *Acheta domesticus* could form a gel under the condition of pH = 7. At a protein concentration of 30% *w*/*v*, the supernatants of *Acheta domesticus* were all capable of forming gels at pH = 7/10.	[84]
Gelation	pH	*Tenebrio molitor*	pH 5.5: The gel relies more on hydrogen bonding, resulting in a more particulate structure with poor homogeneity. pH 7.5: Enhanced electrostatic repulsion promotes protein unfolding, leading to a denser gel with the highest storage modulus.	[117]
Gelation	Treatment method	*Hermetia illucens*	Ultrasonic treatment significantly improved protein gel properties, with the maximum particle size of 245.3 nm, the highest surface hydrophobicity of 617.9, the optimal elastic modulus of 2900 Pa, and the densest microstructure (pore size of 0.54 μm).	[110]
OHC	Treatment method	*Acheta domesticus*	Under PEF treatment conditions, the OHC significantly increased, with a maximum enhancement of 4.13 g oil/g.	[118]
OHC	Treatment method	*Tenebrio molitor*	Under HHP treatment conditions, the OHC of insect hydrolysates doubled, increasing from 1.21 g oil/g to 2.42 g oil/g.	[118]
OHC	Temperature	*Tenebrio molitor*	Temperature was 55 °C, and the OHC was 1.62 g oil/g. Temperature was 75 °C, and the OHC was 1.66 g oil/g. Temperature was 95 °C, and the OHC was 1.74 g oil/g.	[115]
OHC	Temperature	*Sphenarium purpurascens Charpentier*	Temperature was 60 °C, and the OHC was 2.79 g oil/g. Temperature was 90 °C, and the OHC was 2.16 g oil/g.	[114]
WHC	Treatment method	*Tenebrio molitor*	After defatting, the WHC of *Tenebrio molitor* powder increased from 1.24–1.31 g water/g to 1.97–2.02 g water/g.	[116]
WHC	pH	*Gryllus assimilis*	As the pH deviates further from the isoelectric point, the WHC increases. The isoelectric point of cricket protein is 3.85, with a WHC of 1.73 g water/g at pH 5.5 and 1.82 g water/g at pH 7.0.	[119]
WHC	Particle size	*Protaetia brevitarsis*	Particle size was 40 mesh, and the WHC was 4.42 ± 0.01 g water/g. Particle size was 100 mesh, and the WHC was 4.78 ± 0.10 g water/g. Under ultrafine grinding treatment conditions, the WHC was 5.07 ± 0.11 g water/g.	[120]
WHC	pH	*Acheta domesticus*	At pH 4, the WHC was the lowest. At pH 12, the WHC was 0.24–0.26 g water/g.	[96]

Abbreviations: EA, emulsifying activity; ES, emulsifying stability; EC, emulsifying capacity; FC, foaming capacity; FS, foaming stability; HHP, high hydrostatic pressure; PEF, pulsed electric field; OHC, oil-holding capacity; WHC, water-holding capacity.

## 4. Bioactivities of Insect Protein-Derived Hydrolysates and Peptides

Insect protein-derived hydrolysates and peptides have gained attention not only for their nutritional value but also for their diverse bioactivities. Studies have shown that these compounds can exert antioxidant, anti-hypertensive, anti-diabetic, and antimicrobial effects, making them promising functional ingredients for health-related applications.

### 4.1. Antioxidant Activity

Antioxidants inhibit the damage caused by harmful reactive oxygen species or Non-free radical reactive oxygen species. They exert protective effects by shielding cellular components like membrane lipids, proteins, and DNA. This mechanism is vital in preventing and managing numerous diseases [121]. Lee et al. prepared *T. molitor* protein treated with varying ethanol concentrations and evaluated its structural, techno-functional, and antioxidant properties. The results indicated that ethanol treatment altered the techno-functional properties of *T. molitor*, with 20% ethanol yielding the most potent antioxidant activity [122]. Various methods have been reported to enhance the antioxidant activity of insect protein, such as hydrolysis, ultrasound-assisted treatment, chromatographic separation, and glycosylation. Mintah et al. found that ultrasound-assisted enzymatic hydrolysis could enhance the antioxidant activity of *Haplia nitidula* larva protein (HILMP). Ultrasonically treated hydrolysate, prepared at 80 min of enzymatic hydrolysis and 50 °C, displayed the highest hydroxyl radical scavenging activity at 72%. This value was notably higher than the 58% recorded in the control group treated with conventional enzymatic hydrolysis [123]. Francielle et al. demonstrated that enzymatic hydrolysis of black cricket (*Gryllus assimilis*) protein with Flavourzyme^TM^ 500 L, Alcalase^TM^ 2.4 L, and Neutrase^TM^ 0.8 L either alone or in combination significantly improved its antioxidant property, with Flavourzyme^TM^ 500 L showing the highest antioxidant property against DPPH and ABTS radicals [31]. Zielińska et al. demonstrated that heat treatment can improve the antioxidant capacity of peptide components obtained via in vitro gastrointestinal enzymatic digestion of Gryllodes sigillatus proteins. Peptide isolates from Gryllodes sigillatus protein preparations displayed the most excellent anti-free radical activity against ABTS and DPPH, with EC_50_ values of 2.75 and 6.91 μg/mL, respectively. Furthermore, among all isolated peptides, those derived from *T. molitor* protein preparations exhibited the highest Fe^2+^ chelating ability (EC_50_ = 2.21 µg/mL) and the strongest reducing power (0.198) [100]. Vercruysse et al. confirmed that hydrolysates from Spodoptera littoralis exhibited potent antioxidant activity, as measured by the DPPH and FRAP assays [124]. Additionally, Mshayisa et al. reported that binding black soldier fly larvae protein with glucose via the Maillard reaction significantly enhanced its antioxidant activity, especially under increased temperature and time. This enhancement was possibly due to alterations in protein structure and the generation of browning products [125]. These methods provide strong support for utilizing insect proteins as novel functional ingredients in food, and open up new possibilities for developing food additives with antioxidant activity.

### 4.2. Anti-Hypertensive Activity

Hypertension is universally acknowledged as a major risk factor for cardiovascular diseases, stroke, kidney disease, and other health-related disorders. Angiotensin-converting enzyme (ACE) plays a crucial role in the renin–angiotensin system [126], primarily facilitating the conversion of angiotensin I to the potent vasoconstrictor angiotensin II. This conversion triggers a rise in blood pressure through various pathways, including vasoconstriction, aldosterone secretion, and promoting vascular remodeling and fibrosis [124,127]. Therefore, there is a substantial focus on ACE inhibitors as a therapeutic approach for managing hypertension. Researchers are also delving into the ACE inhibitory activity of insects, aiming to uncover the potential health benefits of incorporating insect-based foods in diets for hypertension management [128].

Staljanssens et al. investigated the ACE inhibitory potential of aqueous extracts from three different insect cell lines: S2 (embryo, *Drosophila melanogaster*), Sf21 (ovary, *Spodoptera frugiperda*), and Bm5 (ovary, *Bombyx mori*). The results showed that Half Maximum Inhibitory Concentration (IC_50_) values ranged from 700 to 900 μg/mL, indicating significant biological activity against ACE [129]. Mendoza et al. found that the cricket powder fraction fermented with Lactobacillus NRRL B-50572 for 24 h showed the highest antioxidant activity, along with a 23.47% ACEI inhibition rate, with an IC_50_ of 970 μg/mL [130]. Zhang et al. studied the effects of silkworm pupa peptides on spontaneously hypertensive rats (SHR), conducting short-term and long-term administration experiments. The results found that these peptides significantly reduced the blood pressure levels in SHR, indicating their efficacy in lowering hypertension [131]. Jia et al. subjected silkworm pupa protein (SPP) to ultrasound treatment followed by alkaline protease hydrolysis, and purified the tripeptide Lys-His-Val, which exhibited the highest ACE inhibitory activity with an IC_50_ of 12.82 μM. This suggests that the tripeptide derived from SPP could be a promising candidate as a natural ACE inhibitor for pharmaceuticals or functional food ingredients [132]. Wu et al.found that hydrolysates obtained from silkworm larvae protein isolate (SLPI) digested by gastrointestinal enzymes had vigorous ACE inhibitory activity, with an IC_50_ value (8.3 µg/mL) close to that of the positive control captopril (5.3 µg/mL), suggesting the potential of SLPI as an anti-hypertensive agent through its ACE inhibition property [133]. Stella et al. applied advanced proteomics and metabolomics techniques to investigate the effects of *T. molitor* dietary supplementation on hypertension. The research revealed that *T. molitor* supplementation significantly modulated spontaneously hypertensive rats’ serum proteome and metabolome, normalizing key metabolites and proteins involved in biological pathways associated with blood pressure maintenance. The study demonstrates the potential of *T. molitor* as a beneficial functional food supplement for treating hypertension [134]. These findings pave the way for future exploration of insects as natural ACE inhibitors, offering new avenues and directions for developing novel anti-hypertensive drugs or functional foods.

### 4.3. Anti-Diabetic Activity

Diabetes is a metabolic disorder characterized by high blood sugar levels and insulin dysfunction, leading to complications like retinopathy, nephropathy, neuropathy, obesity, and cardiovascular diseases. Managing diabetes and obesity often involves inhibiting digestive enzymes such as dipeptidyl peptidase IV (DPP-IV), α-amylase, and α-glucosidase. DPP-IV inactivates incretin hormones (GLP-1 and GIP), which promote insulin secretion. Inhibiting DPP-IV can enhance glucose-dependent insulin secretion and reduce postprandial hyperglycemia [135]. However, synthetic β-glucosidase inhibitors may cause gastrointestinal issues, leading to the exploration of natural alternatives like bioactive peptides. The enzymatic hydrolysis of proteins to obtain bioactive peptides from insects was first described in 2005, with ACE-inhibitory peptides identified in several insect species.

Parameswaran et al. investigated the pretreatment of HILMP using multi-frequency sweep ultrasound (SFU mode, 40 ± 2 kHz) combined with Alcalase enzymatic hydrolysis. The reaction was carried out under pH 9.0 and 50 °C conditions for 80 min. The resulting hydrolysates exhibited significantly enhanced antioxidant activity: the hydroxyl radical scavenging activity reached 72.3% (an increase of 24.4% compared to the traditional enzymatic hydrolysis group, *p* < 0.05), and the DPPH and ABTS^+^ radical scavenging rates were elevated to 65.8% and 78.5%, respectively [136]. Rivero-Pino et al. studied the effect of ultrasound treatment and sequential enzymatic hydrolysis on the release of β-glucosidase inhibitory peptides from mealworm protein. The study demonstrated that a 15-min ultrasound pretreatment, followed by 1 h of trypsin hydrolysis and an additional 1.5 h of Subtilisin hydrolysis, improved the subsequent release of bioactive peptides [137]. In another study by Rivero-Pino et al., they used subtilisin, trypsin, and Flavourzyme^TM^ 1000 L to treat mealworms (*T. molitor*) and isolate and identify DPP-IV and α-glucosidase inhibitory peptides. The results indicated that peptides with molecular weights between 500 and 1600 Da exhibited the most vigorous DPP-IV inhibitory activity, with an IC_50_ value of 910 μg/mL [138]. Francielle et al. explored the anti-diabetic potential of enzymatic hydrolysates of protein concentrates from *Gryllus assimilis*. The results indicated that the hydrolysates prepared with a mixture of Flavourzyme^TM^ 500 L and Neutrase^TM^ 0.8 L significantly decreased the activities of both α-amylase and α-glucosidase, thereby enhancing the samples’ potential anti-diabetic properties [139]. In a study by Yoon et al., edible insects, including *T. molitor*, *G. bimaculatus*, and *Bombyx mori*, were hydrolyzed to assess their ability to inhibit α-glucosidase. It was observed that non-hydrolyzed samples exhibited the lowest inhibitory capacity, while samples treated with Flavourzyme and Alcalase showed the highest bioactivity, achieving approximately 40% inhibition of α-glucosidase activity [140]. Consequently, insect protein hydrolysates may offer natural diabetes-fighting properties, paving the way for future development of safer and more potent treatments as functional foods for diabetes with continued research.

### 4.4. Antimicrobial Activity

Many organisms, from fungi to plants and animals, can produce antimicrobial peptides (AMPs). These positively charged small peptides (15–30 amino acids) participate in various defense-related processes, including killing pathogens, binding and neutralizing endotoxins, and regulating immune responses to infections [141]. The function of AMPs has expanded from simple pathogen defense to the precise regulation of host-associated symbiotic microorganisms, such as selectively inhibiting non-symbiotic bacteria to maintain a mutually beneficial relationship between the host and beneficial microbes [142]. AMPs are widespread in eukaryotes but have been studied more extensively in insects [143]. Since the isolation of the first insect AMP from the silkworm in 1980 [144], about 150 AMPs have been isolated from a diverse range of insect species, such as *Acalolepta luxuriosa*, *Apis mellifera*, *Bombyx mori*, *Galleria mellonella*, *Heterometrus spinifer*, and *Holotrichia diomphalia* [145]. The variety of individual AMPs generated by insects is remarkable and species-dependent. Their structure and function can be classified into four classes: insect defensins, cecropins, proline-rich AMPs, and glycine-rich AMPs. The broad-spectrum antibacterial, antifungal, anti-trypanosomal, and anti-leishmanial activities of these insect-derived AMPs present promising prospects for new antibiotic alternatives.

Mylonakis and colleagues emphasized the use of *Caenorhabditis elegans* as a whole-animal model for high-throughput screening to identify AMPs effective against human pathogens, such as *Pseudomonas aeruginosa* and Methicillin-resistant *Staphylococcus aureus*. It was also found that the extracted AMPs, when used in combination with antibiotics, significantly restored the sensitivity of multidrug-resistant pathogens to these drugs [146]. Rahnamaeian and colleagues investigated the synergistic effects of two co-existing insect AMPs (the bumblebee linear peptides hymenoptaecin and abaecin). Their findings indicated that the combined presence of these peptides, even at low concentrations such as 1.25 μM for abaecin, could significantly enhance the bactericidal activity of hymenoptaecin. This insight points to the strategic use of AMP combinations against antibiotic-resistant Gram-negative bacteria [147]. Robles-Fort and colleagues explored the antimicrobial activity of the extended peptide TcPaSK derived from insect defensin three against *Staphylococcus aureus*. The TcPaSK peptide exhibited superior antimicrobial activity with proteomic evidence of downregulated proteins involved in cell growth and tumor progression. It suggests its potential as a multifunctional molecule for novel therapeutic strategies to overcome bacterial antibiotic resistance [148]. Bertrams and colleagues investigated the antibacterial activity of insect-derived AMPs against *Streptococcus pneumoniae*, revealing that defensin one from *Tenebrio castaneum* could inhibit the growth of multidrug-resistant S. pneumoniae in vitro and reduce cytokine production in infected macrophages [149]. Similarly, Rajamuthia et al. studied the in vitro activity of defensin one from *Tribolium castaneum* against methicillin-resistant *Staphylococcus aureus* strains, which enhanced *Caenorhabditis elegans* survival without cytotoxicity to human red blood cells [150]. Makarova and colleagues conducted a 21-day study on the proteomic processes of the immune response of Tenebrio to heat-inactivated *Staphylococcus aureus* infection. The study revealed that AMPs in Tenebrio were expressed at sustained high levels post-infection, forming a persistent antimicrobial environment [151]. Insect AMPs have been extensively studied and show promising prospects for application. By harnessing the synergistic effects of insect AMPs and their effectiveness against antibiotic-resistant bacteria, we can anticipate the development of more effective antimicrobial therapies in the future (Table 4).

**Table 4:** Summary of the effects of different processing methods on the bioactivities of insect protein hydrolysates.

Bioactivities	Source	Processing Method	Influence	Reference
Antioxidant	*Tenebrio molitor*	Ethanol treatment	The 2,2′-Azino-bis(3-ethylbenzothiazoline-6-sulfonic acid) (ABTS) radical scavenging activity of untreated mealworm protein was approximately 34%, whereas that of the protein treated with 20% ethanol reached the highest level (38%).	[122]
Antioxidant	*Hermetia illucens*	Ultrasound-assisted enzymatic hydrolysis	Under the conditions of enzymatic hydrolysis time of 80 min and temperature of 50 °C, the hydrolysate treated with ultrasound has the highest hydroxyl radical scavenging rate, which is 72%.	[123]
Antioxidant	*Gryllus assimilis*	Enzymatic hydrolysis	The enzymatic hydrolysis using Flavourzyme^TM^ 500 L alone exhibited the most remarkable positive effect on the antioxidant properties of proteins. Its IC_50_ values for DPPH and ABTS radical scavenging activities were 455 and 71 µg/mL, respectively.	[31]
Antioxidant	*Gryllodes sigillatus*	Heat treatment	The peptides obtained after the treatment exhibited the highest scavenging activity against ABTS and DPPH free radicals, with EC_50_ values of 2.75 and 6.91 μg/mL, respectively.	[100]
Antioxidant	*Spodoptera littoralis*	Enzymatic hydrolysis	The hydrolysates obtained by simulated gastrointestinal digestion (IC_50_ = 320 μg/mL) and mucosal enzyme digestion (IC_50_ = 211 μg/mL) exhibited strong antioxidant activity in vitro.	[124]
Antioxidant	*Hermetia illucens*	Maillard reaction	The ABTS^+^ radical scavenging activity of *Hermetia illucens* larva-glucose conjugate produced at 90 °C showed significantly higher scavenging activity, which varied with the reaction time (reaching a maximum of 55% at 10 h).	[125]
Antioxidant	*Hermetia illucens*	Ultrasound-assisted enzymatic hydrolysis	The antioxidant activity of hydrolysates obtained by multi-frequency swept-frequency ultrasound (SFU mode, 40 ± 2 kHz) pretreatment combined with Alcalase enzymatic hydrolysis of *Hermetia illucens* larva meat protein (HILMP) was significantly enhanced: the hydroxyl radical scavenging rate reached 72.3%.	[136]
Anti-hypertensive	Grasshopper	Fermentation	Cricket powder fermented with *Lactobacillus* NRRL B-50572 for 24 h exhibited an ACE inhibitory rate of 23.47% and an IC_50_ value of 970 μg /mL.	[130]
Anti-hypertensive	*Gryllus assimilis*	Enzymatic hydrolysis	The peptides obtained from protein hydrolyzed by the binary enzyme mixture of Flavourzyme^TM^ 500 L and Alcalase^TM^ 2.4 L exhibited the highest ACE inhibitory rate, reaching 50.84%.	[139]
Anti-hypertensive	*Gryllodes sigillatus*	Enzymatic hydrolysis	Cricket protein hydrolysates (DH 60–85%) and their digested products exhibit strong ACE inhibitory activity (inhibition rate > 90%, IC_50_ as low as 51 μg/mL), showing potential for improving hypertension.	[152]
Anti-hypertensive	*Musca domestica*	Extraction with water	The water extract of *Musca domestica* larvae exhibits significant ACE inhibitory activity (IC_50_ = 430 μg/mL), demonstrating potential for improving hypertension.	[153]
Anti-hypertensive	*Bombyx mori*	Ultrasonic treatment	Silkworm pupae protein was treated with ultrasonic waves at a power of 410 W/100 mL, followed by hydrolysis with Alcalase for 32 min. The hydrolysate with the highest ACE inhibitory activity (IC_50_ = 4.9 μg /mL) was obtained at a hydrolysis time of 50 min.	[132]
Anti-hypertensive	*Bombyx mori*	Enzymatic hydrolysis	The hydrolysate of silkworm larva protein isolate (SLPI) digested by gastrointestinal enzymes in vitro exhibits strong ACE inhibitory activity, with an IC_50_ value of 8.3 µg/mL, indicating its potential as an anti-hypertensive active ingredient.	[133]
Anti-diabetic	*Tenebrio molitor*	Ultrasonic treatment	Experimental results indicated that after the protein was subjected to 15-min ultrasonic pretreatment (US15) followed by 1.5-h trypsin hydrolysis, the α-glucosidase inhibition rate exceeded 85% and remained consistently high thereafter.	[137]
Anti-diabetic	*Tenebrio molitor*	Enzymatic hydrolysis	Peptides with DPP-IV and α-glucosidase inhibitory activities were isolated and identified from enzymatically hydrolyzed *Tenebrio molitor* protein. The results showed that peptides with molecular weights between 500 and 1600 Da exhibited the strongest DPP-IV inhibitory capacity (IC_50_ = 910 μg/mL).	[138]
Anti-diabetic	*Tenebrio molitor*	Aqueous extract	The hot extract of *Tenebrio molitor* showed strong α-amylase (IC_50_ = 410 μg/mL) and α-glucosidase inhibition (IC_50_ = 7400 μg/mL), while the cold extract was more effective against lipase (IC_50_ = 430 μg/mL).	[154]
Anti-diabetic	*Bombyx mori* *Protaetia brevitarsis* Caelifera *Gryllus bimaculatus* *Tenebrio molitor* *Allomyrina dichotoma*	Cordyceps fermentation	Fermented insects exhibit anti-diabetic effects by promoting glucose absorption.	[155]
Anti-diabetic	*Gryllus assimilis*	Enzymatic hydrolysis	Flavourzyme^TM^ 500 L: Neutrase^TM^ 0.8 L, 1:1—α-amylase inhibition: 55.40 ± 2.93%; α-glucosidase inhibition: 17.07 ± 1.32%; IC_50_: 1990 μg/mL (α-amylase) and 6210 μg/mL (α-glucosidase).	[139]
Anti-diabetic	*Bombyx mori*	Enzymatic hydrolysis	The protein samples hydrolyzed by Flavourzyme and Alcalase exhibited the highest bioactivity, with an α-glucosidase inhibition rate of approximately 40%.	[140]
Antimicrobial	bumblebee	Immunization with *E. coli* and isolation of peptide from the hemolymph	Combination treatment (low-dose hymenoptaecin with 1.25 μM abaecin) resulted in a statistically significant reduction in CFU counts compared with either peptide alone (*p* < 0.05)	[147]
Antimicrobial	*Galleria mellonella*	Immunization with viable *E. coli* D31 and isolation of peptide from the hemolymph	Eight defensive peptides were isolated and identified from the hemolymph of *Galleria mellonella* larvae under immune challenge, five of which are newly discovered with diverse antimicrobial activity profiles, and the Gm defensin-like peptide shows the strongest activity.	[147]
Antimicrobial	*Apis cerana*	Recombinant proteins are expressed in the baculovirus-insect cell system and purified by Strep-tag affinity chromatography.	*Apis cerana* venom serine protease inhibitor inhibits serine proteases (trypsin IC_50_ = 1.37 ± 0.20 μg/mL; proteinase K IC_50_ = 1.14 ± 0.07 μg/mL; plasmin IC_50_ = 2.24 ± 0.51 μg/mL), and exerts broad antimicrobial activity (*B. thuringiensis* MIC_50_ = 8.47 ± 0.67 μg/mL; *E. coli* MIC_50_ = 16.80 ± 1.26 μg/mL; *B. bassiana* IC_50_ = 9.80 ± 1.19 μg/mL).	[156]
Antimicrobial	*Tribolium castaneum*	The TcPaSK peptide was chemically synthesized based on an extended sequence of *Tribolium castaneum* insect defensin 3.	The minimum inhibitory concentration (MIC) range of TcPaSK against *Staphylococcus aureus* is 16–32 µg/mL, indicating its effective inhibition of bacterial growth. Notably, this concentration range is significantly lower than the toxic concentration towards mammalian cells (>100 µg/mL), which demonstrates the peptide’s specific targeting of bacteria.	[148]
Anti-microbial	*Tribolium castaneum*	*Tribolium castaneum* Defensin 1 was synthesized by solid-phase synthesis.	*Tribolium castaneum* Defensin 1 at a concentration of 12.5 μg/mL could increase the survival rate of nematodes infected with *S. aureus* from 22% to 87%.	[150]

## 5. Allergenicity of Insect Proteins

### 5.1. Clinical Reactions to Allergens

**Figure 1:**
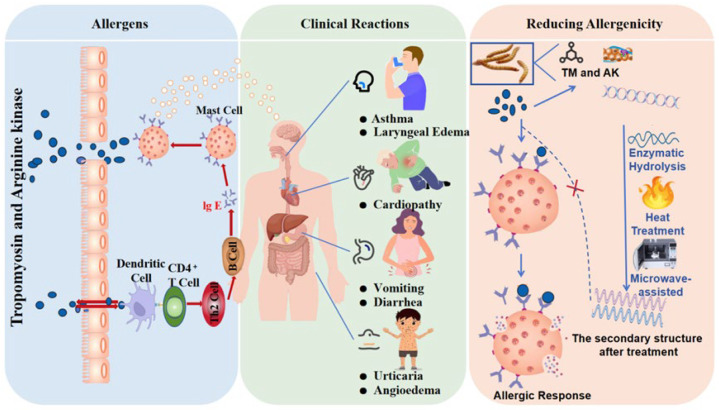
Insect protein allergens, clinical allergic reactions, and approaches to reduce allergenicity.

As emerging sustainable protein sources, insect proteins have raised allergenicity-related clinical safety concerns, becoming a key focus at the intersection of food science and immunology. The clinical manifestations of insect protein allergy are not merely scattered case reports but present a recognizable pattern of immunoglobulin E (IgE)-mediated hypersensitivity reactions. As shown in Figure 1, the illustration details allergen-mediated allergic reactions, associated clinical symptoms, and allergenicity-reducing methods. Current evidence indicates that insect allergy shares pathological mechanisms with crustacean and mite allergies, primarily driven by IgE cross-reactivity to conserved proteins such as tropomyosin (TM) and arginine kinase (AK) [157]. Due to the high homology of these proteins across arthropods, individuals allergic to crustaceans are at elevated risk, with epidemiological studies reporting an allergy prevalence of 3–8% among insect consumers [158]. For example, a survey conducted in Laos involving 1059 individuals who regularly consumed insects reported an allergic reaction rate of 7.6%. Although the specific insect species responsible for sensitization were not definitively identified, bedbugs and grasshoppers were listed as potential allergenic sources. Importantly, no fatal outcomes were reported [159]. These findings align with the European Food Safety Authority (EFSA)’s assessment of allergenic risks associated with *T. molitor* [158].

The clinical manifestations of insect protein allergies vary widely. Mild cases predominantly present with dermatological symptoms such as urticaria and angioedema, while moderate to severe reactions may involve the respiratory tract (e.g., asthma, laryngeal edema) and gastrointestinal system (e.g., vomiting, diarrhea) [158]. The duration of symptoms can range from several minutes to up to six hours and is strongly influenced by factors such as the amount of insect protein ingested, the method of processing, and the individual’s immunological status [157]. The spectrum of these reactions indicates that insect allergy constitutes a potentially serious health hazard. Although reports of severe anaphylactic reactions are relatively fewer compared to some major allergens such as peanuts, the prevalence of systemic reactions (involving the respiratory and gastrointestinal tracts) underscores a significant barrier to consumer safety and acceptance. The dose dependency and variability in the onset of symptoms further complicate risk assessment for both consumers and regulatory authorities. Although systemic allergic reactions, such as hypotension, have been documented, fatal cases have not yet been reported. A rare case involving sago worm ingestion complicated by Takotsubo cardiomyopathy has been described, suggesting that insect protein may trigger systemic inflammatory cascades with potential cardiovascular implications [160]. Ji et al. documented a case of anaphylactic shock in a French male with a history of allergic rhinitis following his first consumption of *Bombyx mori* larvae. Furthermore, a review of 13 Chinese patients who experienced severe allergic reactions after consuming silkworm pupae for the first time-despite having no prior history of anaphylaxis-highlighted the potential of insect protein to induce both primary sensitization and cross-reactive immune responses [161].

Another study reported five cases of allergic reactions to *Cordyceps* larvae, primarily presenting with widespread urticaria, with some patients also exhibiting conjunctivitis and rhinorrhea. Immunological analyses revealed cross-reactivity between *Cordyceps* larvae and silkworm pupae, with specific IgE antibodies against both detected in four patients. This suggests that the allergic response may involve IgE- and non-IgE-mediated pathways [162]. These case studies reveal two key insights: first, primary sensitization to insects can occur even in individuals with no prior allergic history. Second, cross-reactivity is a major driver of allergic reactions, particularly in individuals already sensitized to crustaceans or dust mites. This dual sensitization pathway significantly expands the scope of the potentially at-risk population, extending it beyond those who intentionally consume insects. The broad spectrum of clinical manifestations and variability among affected populations underscores the complexity of insect protein allergy and its public health relevance. These findings highlight the urgent need to investigate the specific allergenic components of insect proteins to evaluate their safety for human consumption fully. The occurrence of clinical allergic reactions is closely linked to specific allergens in insect proteins. Identifying the types and characteristics of these allergens is crucial for risk management.

### 5.2. Allergens in Edible Insect Proteins

The allergenic potential of edible insects is primarily attributed to their possible cross-reactivity with other arthropods, particularly crustaceans. The mechanism underlying such cross-reactivity is rooted in the high amino acid sequence identity and structural homology of certain allergen families in arthropods [163]. IgE antibodies produced against a specific allergen from one source (e.g., TM from shrimp) may fail to distinguish this allergen from its highly similar homologs from another source (e.g., TM from crickets), thereby leading to clinical cross-reactivity. Crustaceans are among the most common food allergens in Western countries, and recent phylogenetic analyses suggest a closer taxonomic relationship between insects and crustaceans than previously recognized, thereby substantiating the plausibility of immunological cross-reactivity between them [164,165]. Notably, cross-reactivity between non-edible insects (e.g., cockroaches) and crustaceans has been extensively studied, with TM and AK identified as the major pan-allergens responsible for IgE-mediated cross-reactivity across arthropods [157,166,167,168]. A series of experimental studies have demonstrated that these proteins can elicit allergic responses in murine models, including significantly elevated serum histamine and IgE levels, along with a Th1/Th2 cytokine imbalance in cultured splenocytes and mesenteric lymph node cells [169,170,171].

TM, a member of the TM family, is a muscle-associated protein that plays a key role in muscle contraction by interacting with myosin and actin filaments. Its high conservation across invertebrate species, surface exposure, and stability make it a primary target for IgE recognition and a classic pan-allergen. It is ubiquitously expressed in muscle and non-muscle cells and has been identified in various allergenic sources such as dust mites, crustaceans, moths, and cockroaches [115]. AK, an enzyme belonging to the ATP: guanidino-phosphotransferase family, is a key isoform within the phosphagen kinase family, including vertebrate allergens such as creatine kinase. Similarly, the functional conservation of AK across different species endows it with a high degree of structural similarity, which also explains the mechanism underlying its role as another major cross-reactive pan-allergen [172]. AK catalyzes the reversible transfer of a phosphate group between ATP and arginine, thereby maintaining cellular energy homeostasis [173]. The molecular characteristics and high sequence homology of these allergenic proteins in insect species and other arthropods constitute a fundamental basis for the allergenicity of insect-derived proteins. This highlights the necessity for thorough risk assessments in sensitized populations and the development of optimized processing strategies to mitigate potential allergenicity.

Multiple isoforms of TM and AK have been identified across various arthropod species. 27 TM isoforms and 17 AK isoforms from arthropods have been recognized as food allergens. Recently, several allergenic proteins have been identified in edible insect species. For instance, Jeong et al. characterized TM from *Bombyx mori* pupae as a pan-allergen among invertebrates. The TM gene was cloned via reverse transcription PCR and heterologously expressed in *Escherichia coli*. ELISA assays demonstrated that sera from 53.3% (8/15) of patients allergic to *Bombyx mori* showed IgE binding to the recombinant TM. However, inhibition assays revealed limited IgE reactivity (<10%) with the total pupa extract [174].

Verhoeckx et al. investigated *T. molitor* proteins for their IgE cross-reactivity with sera from individuals allergic to dust mites and crustaceans using immunoblotting and basophil activation tests. Additionally, in vitro digestion assays with gastric pepsin indicated that TM and AK retained structural stability and allergenicity, confirming their sensitization potential [175]. Lamberti et al. assessed the IgE cross-recognition of proteins from five edible insect species (mealworm, buffalo worm, silkworm, cricket, and locust) after thermal processing (boiling and frying). Their findings identified TM as a significant cross-reactive allergen in patients sensitized to dust mites and crustaceans [176]. Kamemura et al. identified 10 allergenic proteins in crickets, among which TM and its isoforms showed strong cross-reactivity with sera from crustacean-allergic patients [177].

Furthermore, Wang et al. reported that the major allergen in locusts is a hexamerin-2-like protein, which exhibited significant IgE binding with sera from locust-allergic individuals [168]. Sookrung et al. demonstrated that AK is a significant allergen in Periplaneta americana, reacting with IgE in the sera of all tested cockroach-allergic patients [167]. These findings collectively indicate that the allergenic proteins present in insect species share high structural and functional similarity with those in crustaceans and may retain their allergenic potential even after food processing. Such characteristics pose critical challenges for the safe application of insect proteins in the food industry.

Most existing studies adopt a candidate allergen verification strategy (e.g., targeting TM and AK). While this approach enables the rapid identification of known cross-reactivity risks, it may overlook novel, insect-specific allergens that lack homologs in well-studied taxa [178]. To systematically map the allergen profile of edible insects, future research is likely to focus on employing unbiased omics strategies (e.g., proteomics combined with IgE immunoprofiling of sera from allergic patients), thereby providing a comprehensive target basis for subsequent processing or desensitization strategies [179,180].

### 5.3. Reducing Allergenicity

In food science, reducing or eliminating the allergenicity of food allergens has long been a central research focus. A key criterion for evaluating the effectiveness of such efforts lies in minimizing the IgE-binding capacity of allergens. Modifications to linear and conformational epitopes during food processing can reduce the IgE-binding ability of allergens, thereby mitigating IgE-mediated allergic responses [166,181]. The following examples illustrate the diverse and complex aspects of understanding the efficacy and limitations of food processing techniques to reduce or eliminate food allergens in edible insects.

Enzymatic hydrolysis is the most extensively studied method for reducing the IgE-binding potential of insect proteins and has been widely applied to mitigate the allergenicity of insect proteins. Studies have demonstrated that insect proteins can be hydrolyzed into smaller peptides through the appropriate selection of enzymes, which exhibit lower immunogenicity compared to intact proteins [152]. Additionally, specific enzymatic treatments can disrupt the three-dimensional structure of allergenic proteins, thereby reducing the probability of IgE antibody binding [182]. Hall et al. conducted enzymatic hydrolysis of cricket proteins using Alcalase, and their findings revealed that when the degree of hydrolysis ranged from 60% to 85%, the IgE reactivity to TM was nearly eliminated or extremely low [152]. Giulia et al. evaluated the allergenicity of *T. molitor, H. illucens*, and their hydrolysates through shotgun proteomics, computational prediction, and immunoblotting, and found that hydrolysis using *Bacillus licheniformis* protease (1%, 60 °C, pH 7.5) could reduce the immunoreactivity of insect proteins [179]. He et al. demonstrated that treating *T. molitor* proteins with pepsin and trypsin significantly reduced their allergenicity. Pepsin degraded high-molecular-weight proteins (>33 kDa) into low-molecular-weight fragments (<25 kDa), while trypsin eliminated proteins >70 kDa. Pepsin appeared more effective in reducing allergenicity, indicating that gastrointestinal enzymatic hydrolysis can effectively lower the allergenic potential of insect proteins [183]. In addition, Boukil et al. studied the application of high hydrostatic pressure (HHP) combined with alkaline protease or pepsin to hydrolyze *T. molitor* proteins, thereby enhancing in vitro digestion, especially of allergenic proteins. Their results showed that certain pressure conditions combined with pepsin improved the in vitro digestion of major allergens [184]. However, enzymatic hydrolysis also has limitations. The hydrolysis process may generate new peptide fragments with unknown allergenic potential, and the extent to which allergenicity is reduced depends mainly on the type of enzyme, the degree of hydrolysis, and the substrate source [183,185]. For example, mealworm hydrolysates showed almost no IgE binding with TM in LM. However, the same treatment for BSF retained considerable IgE reactivity, indicating species-dependent residual allergenicity remains a significant issue [179]. Over-hydrolysis can also impair the functional properties of proteins or negatively affect the sensory attributes of products (such as taste and bitterness), thereby limiting consumer acceptance [186].

Thermal processing represents one of the most straightforward approaches to reducing the allergenicity of insect proteins. Its advantages lie in inducing protein denaturation and altering the secondary structure of insect proteins—specifically reducing α-helices and β-sheets. This structural modification may result in the masking of epitopes and enable the partial degradation of major allergens such as TM and AK, thereby significantly diminishing IgE-binding capacity [183,187]. For example, Lee et al. reported that the IgE-binding capacities of TM and AK from *T. molitor* were significantly reduced after heat treatment. However, due to the high thermal stability of these proteins, residual antigenicity persists [188]. Broekhoven et al. examined the effects of boiling and frying on the IgE cross-reactivity of proteins from *T. molitor*, *Alphitobius diaperinus*, and *H. illucens*. They found that while heat treatment reduced cross-reactivity, it did not eliminate it [176]. Cristina et al. further evaluated the effect of heat treatment on the IgE cross-reactivity of proteins from *T. molitor*, *A. diaperinus*, silkworms, crickets, and locusts, finding that changes in protein solubility influenced IgE-binding ability, with effects varying depending on protein type, insect species, and processing method [189]. In summary, although thermal processing can partially reduce the allergenicity of insect proteins, different proteins and species exhibit varied responses to such treatment. Moreover, thermal processing has certain limitations. It may fail to eliminate allergenicity, as some protein fragments, due to their thermostability, can still bind to IgE. Excessive heating can also impair nutritional value (e.g., lysine loss via the Maillard reaction) and functional properties such as solubility and digestibility, while inducing undesirable sensory changes like off-flavors and browning [190]. The variable success of thermal processing underscores the remarkable stability of core insect allergens like TM. This inconsistency suggests that thermal treatment alone is insufficient to guarantee safety for allergic consumers and may be more suitable as a preliminary step in a combined processing chain, rather than a standalone solution.

Studies have indicated that certain protein fragments of specific sizes possess heat and enzyme resistance properties, such that the risk of residual allergenicity may persist following enzymatic hydrolysis and thermal processing [183]. Thus, optimizing the methodologies in response to these circumstances is currently necessary. Microwave-assisted hydrolysis is an emerging novel technology in recent years. It enables rapid heating, disrupts protein folding states, breaks disulfide bonds/hydrophobic aggregates, and induces protein unfolding. This process facilitates easier access of enzymes to allergenic epitopes, enhances hydrolysis efficiency, and reduces the reactivity of proteins with the immune system [191,192]. Hall et al. extracted TM from crickets and compared microwave-assisted hydrolysis and simple heat treatment. Their experiments revealed that microwave-assisted enzymatic hydrolysis of cricket proteins significantly enhanced their biological activities (such as DPP-IV and ACE inhibitory effects) while reducing IgG-binding capacity to major allergens (e.g., TM). Raman spectroscopy indicated structural changes in the amide I and S-S bond regions, which might be associated with reduced immunoreactivity [186]. In subsequent studies, combining enzymatic hydrolysis and microwave heating, coupled with immunoinformatics and proteomics analyses, significantly reduced the number of intact epitope regions in TM, thereby decreasing IgE and IgG reactivity [193]. Dong et al. treated actin using microwaves at 1000 W and 2.45 GHz for 15 min at 125 °C, resulting in a 75% reduction in allergenicity. This reduction was associated with changes in secondary structure, including decreases in α-helices and turns and an increase in β-sheets, which reduced the likelihood of IgE binding in allergic individuals [185]. However, microwave-assisted hydrolysis still has limitations. High microwave power or prolonged treatment may adversely affect nutritional quality, functional properties (such as solubility, foaming capacity, and gelation property), or sensory characteristics (including flavor and color). Furthermore, in industrial food production, this technology’s scale-up application and process control (e.g., temperature uniformity, safety, and cost) are non-trivial [194,195]. Moreover, in the current stage, it is difficult for such technology to achieve large-scale application and economic feasibility in food raw materials with meager profits [196].

A comparative analysis of allergen reduction strategies reveals a distinct trade-off among efficacy, practicality, and cost. Despite its remarkable effectiveness, Enzymatic hydrolysis is hindered by economic costs and challenges related to sensory quality. Conventional thermal processing, while economically feasible, often yields suboptimal results due to the intrinsic stability of allergens [183]. Emerging technologies such as microwave treatment and HHP enable precise processing but suffer from limited scalability [184]. This analysis suggests that the most promising avenue for reducing insect allergenicity in the future may lie in the development of tailored combinatorial processing approaches (e.g., mild thermal pretreatment combined with targeted enzymatic hydrolysis), which maximize epitope destruction while minimizing damage to functional properties and sensory quality. There remains a significant knowledge gap in translating these in vitro findings to in vivo efficacy and safety [197]. Therefore, future research should prioritize clinical trials involving sensitized populations.

## 6. Application of Insect Protein

### 6.1. The Application of Insect Protein in Food

**Figure 2:**
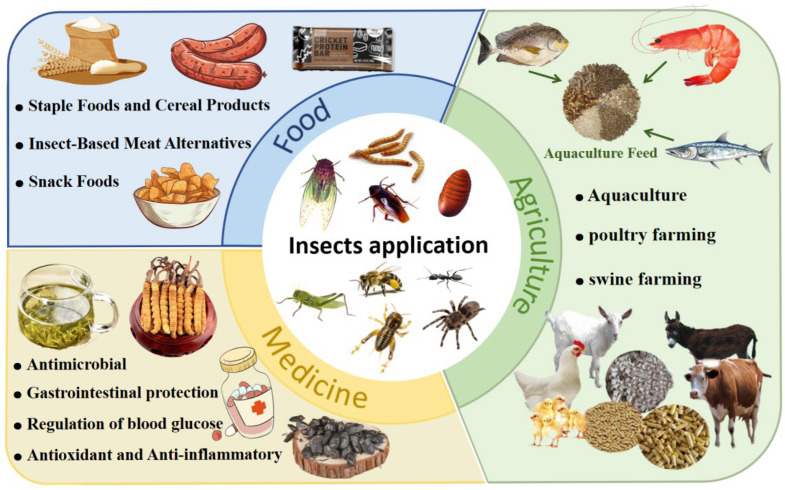
Applications of insect protein.

Insect protein, rich in high-quality EAAs, surpasses conventional animal protein in nutritional value and has emerged as a strategic resource for addressing global food security challenges [198]. Since Meyer-Rochow proposed developing insects as food in 1975 [199], this field has seen significant progress. However, consumer acceptance of insect-based products is still constrained by sensory expectations—most consumers display psychological resistance to whole insect forms, while exhibiting greater tolerance toward invisibly incorporated forms [200,201]. To address this, the food industry has leveraged innovative processing technologies to convert insect proteins into tasteless or hypoallergenic ingredients and integrate them into diversified product matrices, offering “sensory-friendly” solutions [202]. Figure 2 introduces the applications of insect proteins in different fields.

Insect protein has achieved diversified and innovative applications in the global food industry. In the staple and cereal products domain, African researchers developed a high-protein porridge (SOR-Mite) by combining termite flour with sorghum, effectively combating child malnutrition [203]. Chutima et al. extracted protein from four edible insects (*A. domesticus*, *G. bimaculatus*, *Holotrichea* sp., and *Gryllotalpa orientalis*) for flavored rice noodle production. Their findings showed that adding 2–8% IPC improved the noodles’ nutrition and texture, with IPC as a stabilizer or thickening agent [79]. Kowalski et al. demonstrated that insect flour can enhance the nutritional value of wheat bread; specifically, 10% inclusion increased the lysine amino acid score (AAS) [204]. Osimani et al. reported that incorporating cricket powder (*A. domesticus*) into wheat flour improved the bread’s protein content, essential amino acid profile, and fatty acid composition, while maintaining good sensory acceptance [205]. Machado further validated that cricket powder can be an alternative protein source in gluten-free bread formulations [206]. In the snack food sector, the Mexican product “Buqadilla,” combining mealworms and chickpeas, successfully entered the European market. A biscuit enriched with 10% cricket powder was developed in Kenya, receiving above-average sensory ratings and providing key nutrients [207]. Akande et al. confirmed that insect protein can replace skim milk powder in high-energy biscuits; biscuits made from locust powder and silkworm pupae powder (SWP) were rich in protein and vitamins A and C, showed good sensory qualities, and met microbial safety standards [208]. Ortolá et al. further confirmed that powders derived from *T. molitor* and *Alphitobius diaperinus* enabled biscuits to meet “high-protein” labeling standards [209]. García-Segovia et al. found that adding *Alphitobius diaperinus*, *T. molitor*, and pea protein powders enhanced the nutritional value of extruded snacks while maintaining their physicochemical properties [210]. Regarding meat alternatives and fermented foods, Stoops et al. developed minced meat products with *T. molitor* larvae using modified atmosphere packaging to extend shelf life while retaining palatability [211]. Silkworm pupae powder was used in Frankfurt sausage production, reducing cooking loss and improving texture [212]. Additionally, *T. molitor* larvae, defatted insect biomass, and cofermentation with *Aspergillus oryzae* and *Bacillus licheniformis* were used in soy sauce fermentation, which not only enhanced flavor but also improved nutritional value [213].

Over the past decade, more than 250 insect-protein-based products have reached commercialization. The rise of innovative dietary trends, the proliferation of hybrid meat products, widespread media exposure, and increased market accessibility have all driven rapid growth in the insect protein sector, highlighting its vast potential in the food industry.

### 6.2. The Application of Insect Protein in Medicine

In traditional medicine, insects have long played an important role in many countries. In Nigeria, crickets (*Brachytrupes membranaceus*) are a food source and an essential ingredient for promoting cognitive development and in prenatal and postnatal care [214]. In terms of medicinal properties, insects have been used as nutritional foods in some countries for a long time [211]. In Asia, beetles (*Ulomoides dermestoides*) are commonly used as alternative treatments for asthma, arthritis, and tuberculosis [215]. In Brazil and India, cockroaches and ants are used to treat asthma, often consumed as tea [216]. The indigenous knowledge accumulated from these traditional applications of insect medicinal value has provided valuable clues and insights for the bioprospecting of modern drug compounds.

With the advancement of modern scientific research, many in vitro experiments and animal model studies have demonstrated that insect proteins possess various medicinal effects. Consumption of edible insects offers gastrointestinal protection, antioxidant and anti-inflammatory activities, antimicrobial properties, immune modulation, regulation of blood glucose and lipid levels, blood pressure reduction, and a decrease in the risk of cardiovascular diseases [217,218,219]. As a result, numerous studies have explored the clinical applications of insects. For instance, Liao et al. extracted a protein–polysaccharide complex from the American cockroach, which serves as a bioactive substance for wound healing in diabetic mice and accelerates the healing process of acute wounds [220]. Hongyan M et al. found that bioactive substances from the American cockroach can regulate the PI3K/Akt pathway by reducing the relative expression of PI3K and *p*-Akt proteins, thus inhibiting the growth of liver cancer cells [221]. Furthermore, studies by Yu et al. revealed that oral consumption of SWP significantly reduced total triglycerides and low-density lipoprotein cholesterol, thereby improving atherosclerosis [222]. Additionally, studies have shown that SWP lipids benefit various cardiovascular diseases, offering new ideas and potential therapeutic approaches for treating cardiovascular diseases [223,224].

### 6.3. The Application of Insect Protein in Agriculture

In aquaculture, insect feed has been successfully scaled as an alternative to fishmeal. Regarding substitution ratios and effectiveness, in African catfish feed, defatted housefly larva meal can completely replace fishmeal without affecting growth performance. Replacing fishmeal with insect protein in feed can enhance the protein digestibility of farmed animals [225]. For marine fish such as Atlantic salmon, the addition of insect meal should be controlled at ≤25% to avoid deficiency in long-chain omega-3 fatty acids (EPA/DHA) [226]. In terms of functional improvements, adding black soldier fly larvae meal can reduce the cost of tilapia feed by 30% and increase growth rate by 15% [227]. Furthermore, replacing fish oil with yellow mealworm oil optimizes the fatty acid profile of the liver [228]. However, complete fishmeal replacement remains challenging, leading to decreased palatability and amino acid imbalances, which require formulation adjustments, such as adding algae to supplement DHA [229].

In poultry farming, the application of insect protein has achieved a dual improvement in both “nutrition and quality.” In terms of growth performance, replacing 10–20% of soybean meal with defatted housefly larva meal can increase the daily weight gain of broilers by 8% and improve feed conversion ratio by 12% [230]. Regarding meat quality regulation, replacing traditional fats with yellow mealworm oil significantly enhances chicken meat tenderness and juiciness [231]. Additionally, adding cricket powder increases the protein content of breast muscle by 15%. Furthermore, “soy-free” insect-based feed aligns with EU organic certification requirements, helping to increase the product’s premium market value [232].

The current research scale on insect protein is relatively small in pig farming, but its unique advantages have been demonstrated in piglet feed. Regarding protein replacement, black soldier fly larvae meal can completely replace soybean protein and fat in nursery pig diets, reducing diarrhea rates by 40% [233]. Regarding functional applications, replacing fishmeal with cricket powder maintains gut microbiota balance and reduces reliance on antibiotics [234]. From an economic perspective, insect feed costs are 18% lower than the corn–soybean meal system, making it particularly suitable for resource-limited regions [235].

In regions like Sub-Saharan Africa, the localized application of insect feed highlights its flexibility. With minimal processing, feeding live termites or locusts directly can reduce poultry farming costs by 50% [227]. In mixed-use applications, combining garden snails with agricultural waste as co-produced feed yields an energy density of 4.5 kcal/g, comparable to commercial feeds [230]. A circular model has also been developed, where black soldier flies can convert kitchen waste into protein feed, forming a “waste-insect-livestock” closed-loop system [236].

In conclusion, insect protein shows excellent development potential and application value in agriculture, particularly aquaculture, poultry farming, pig farming, and localized practices in small-scale farms. With continued research and technological improvements, insect protein is expected to provide higher-quality, more sustainable feed solutions for agricultural production.

### 6.4. Insect Quality and Consumer Acceptance

Consumers’ acceptance of insect-based foods largely depends on their perception of product quality, which involves multiple aspects such as nutrition, texture, safety, and the surrounding environmental context. Recent studies have repeatedly shown that four quality dimensions play a key role: sensory characteristics (taste, odor, texture, and appearance), product form and presentation, safety and trust markers (including label accuracy and microbial indicators), as well as environmental factors such as price, purchase convenience, and cultural familiarity. These dimensions interact with each other: for instance, even if insect protein has high nutritional value, consumers may not accept it if its taste or appearance is repulsive; whereas clear information on the safety and traceability of origin can alleviate such resistance and encourage consumers to be willing to try it [237].

Sensory quality remains the most immediate factor influencing consumers’ repeat purchase behavior toward insect-based products. A large body of evidence indicates that product presentation is critical in determining initial willingness to try. In Western markets, consumers show significantly higher willingness to attempt insect-based products when insect ingredients are incorporated into familiar food matrices such as pasta and protein bars, rather than being presented in recognizable whole-insect forms [238]. This is corroborated by findings in Serbia, where nearly half (49.4%) of respondents were willing to consume processed insect-containing foods with invisible insect components. In contrast, only approximately 25.4% expressed willingness to consume intact insects [239]. This implies that product development must balance nutritional enhancement with sensory optimization. For instance, some studies have demonstrated that when cricket flour is added to cookies at a proportion of 10–20%, the hedonic ratings of the products are comparable to those of traditional counterparts; however, higher substitution rates often reduce palatability due to negative changes in texture and color [240]. Therefore, identifying the optimal substitution threshold is key to achieving market success.

Labeling and presentation are potent tools to modulate the psychological barriers associated with entomophagy. Packaging design, visual cues, and wording choices exert an influence on consumers’ initial approach behaviors: neutral or benefit-focused language (e.g., “sustainable protein” or “novel protein”) as well as attractive and non-threatening packaging designs can enhance consumers’ purchase propensity [241]; in contrast, explicit emphasis on the insect origin may reduce the purchase intention of consumers who are initially unfamiliar with or averse to insect-based foods [242]. Transparent information regarding allergens and ingredient sources is a prerequisite for building consumer trust. For instance, DNA barcoding analyses conducted in the UK market have revealed discrepancies between the labeled content and actual composition of certain insect products, undermining consumer trust [243]. Therefore, label strategies must strike a proper balance: on the one hand, they should adopt consumer-friendly expressions to reduce psychological barriers, and on the other hand, they must ensure information transparency to gain consumer trust.

Cultural background and region are also closely associated with the strength of consumers’ purchase intention. A cross-national survey (covering 14 countries) has revealed significant differences in people’s perceptions of edible insects’ nutritional value and health effects, and a higher level of knowledge reserve is correlated with greater acceptance [218]. For instance, younger consumers who reside in urban areas and possess environmental awareness exhibit higher acceptance, particularly when the products are marketed with sustainability as a selling point [244]. However, significant differences exist across regions: European consumers demonstrate a stronger aversion to insect-based foods, whereas most respondents from Southeast Asia perceive insect-based foods as everyday food items [245]. A study conducted in Serbia found that familiarity with entomophagy and previous experience consuming insect-based foods are important predictors of purchase intention; in this sample, the predictive role of age and educational level was relatively weak. Similarly, in labeling experiments, many participants with lower food neophobia and a higher tendency to seek variety consumed insect-labeled products earlier than those with higher neophobia [242].

Safety and price factors further influence acceptance. Microbiological safety and allergenicity remain concerns for consumers, especially in regions with underdeveloped regulatory frameworks [246,247]. Cost competitiveness also poses a barrier: insect protein products are generally more expensive than conventional animal proteins, which limits their large-scale development in mainstream markets [248]. Reducing production costs through technological innovation and ensuring unified safety standards is necessary to address these challenges.

In conclusion, the quality of insect-based foods influences consumers’ acceptance through a sequence of links, ranging from the inherent attributes of the product itself (sensory properties, nutrition, safety) to the information conveyed by packaging and labeling, and further to cultural contexts and individual experiences [249]. To transform insect protein from a niche choice into a food product accepted by the general public, the industry and regulatory authorities must develop strategies jointly. These strategies must ensure favorable sensory experiences and rigorous safety testing and facilitate communication through transparent and appropriate approaches [246]. Only in this way can consumers be converted from being merely curious to making repeated purchases, and insects as a sustainable food source can gain long-term recognition.

## 7. Challenges and Prospects

### 7.1. Legislative Challenges

Legislative restrictions (such as standards, labeling, and other regulatory tools) and safety are crucial factors to consider for edible insects to become part of the global food market [250,251]. The legislation on insect protein shows significant regional differences worldwide, and this fragmented regulatory landscape poses multiple challenges for the industry’s development.

As a pioneer in the industrialization of insect protein, the European Union has demonstrated a dynamic approach to legislation. In response to the BSE crisis, the “EU Feed Ban” (EC No. 999/2001) [252] was issued in 2001, which banned the use of insect protein in livestock and poultry feed. However, with technological advancements and improved risk assessments, the 2017 amendment (EC No. 893/2017) [253] marked a policy breakthrough by allowing seven insect species (including black soldier fly, yellow mealworm, and others) to be used as raw materials for aquaculture feed, as well as permitting the use of insect fats and whole insects in pig and poultry feed, although highly processed protein remains strictly restricted. More recently, Regulation (EU 2021/1925) [254] expanded the list by including *Bombyx mori* as an approved species for processing into animal protein for feed, reflecting the continuous regulatory progress in Europe. In the food sector, insects are covered by the “Novel Food Regulation” (EC 2015/2283) [255], which requires systematic evaluations by the EFSA on toxicity, allergenicity, and other factors. The entire production process must comply with seven core regulations, including the “General Food Law” (EC No. 178/2002) [256], the “Feed Hygiene Regulation” (EC No. 183/2005) [257], and the mandatory implementation of the HACCP system to control microbial contamination and heavy metal residues [258].

In North America, a differentiated regulatory landscape exists. The U.S. FDA implements tiered management under the “Food Safety Modernization Act” (FSMA). Insects farmed for human consumption are exempt from PCHF regulation after primary processing, but wild insects and insects used for animal feed are strictly prohibited from entering the human food chain. Although Section 201(f) of the “Federal Food, Drug, and Cosmetic Act” recognizes edible insects as food, the lack of specific production standards means that companies must self-certify compliance through general clauses like GMP certification, microbiological testing, and allergen labeling (such as shellfish allergy warnings) [198,259]. In Canada, progressive legislation has been implemented, with CFIA approving black soldier fly as a protein source for aquaculture and poultry feed, providing limited access for the industry [260].

In Southeast Asia, Thailand, as the global leader in cricket farming, has set a precedent by issuing the world’s first national standard for insect farming (TAS 820-2017), covering the entire process from breeding and hygiene control to product traceability. This standard is implemented by the Thai FDA, ensuring export quality supervision and providing a legislative model for developing countries [261].

Currently, the global insect protein regulatory system is highly fragmented. Over 60% of countries have yet to establish specific regulations, with traditional consumption areas (such as Africa and South America) relying on non-standardized artisanal production, and the industrialization process being hindered by a lack of hygiene standards. Emerging markets (such as Southeast Asia) may have a consumer base but lag in testing systems and risk assessment capabilities. Although international organizations like the “Insect Protein for Food and Feed Platform” (IPIFF) are working to promote global standardization, the regulatory unification process is slow. This legislative lag increases compliance costs for businesses, especially start-ups, and creates a barrier [261]. In the future, multinational collaboration will be necessary to improve the legal framework, balancing safety regulation with industry innovation, and clearing obstacles for the global application of insect protein.

### 7.2. Future Prospects

As a promising new protein resource, insect protein has shown broad development prospects after extensive research and exploration of its nutritional composition, processing technology, and safety. Despite facing numerous challenges in its development, with further research and technological innovation, insect protein is expected to achieve significant breakthroughs in various fields.

Regarding processing technologies, there remains a significant gap in knowledge concerning methods that effectively optimize both protein yield and purity. Moreover, most existing studies are confined to laboratory-scale investigations, limiting their practical applicability [262,263,264,265]. Future studies should prioritize the optimization of processing parameters for insect protein isolates intended for food applications, aiming to achieve desirable functional properties while maintaining cost-efficiency and environmental sustainability. This will help improve the quality of insect protein and reduce production costs, facilitating its widespread use in food and feed industries. For instance, although the non-denaturing drying methods commonly used in laboratories are too costly for industrial-scale application, research could lead to the development of more cost-effective drying technologies that maintain the protein’s functional properties [266].

From the perspectives of production scale and efficiency, expanding production and improving efficiency are key challenges for the insect protein industry. Currently, insect farming is mainly conducted on a small scale, insufficient to meet the growing market demand. Future efforts should focus on developing efficient large-scale farming systems, such as vertical farming methods and automated monitoring systems. Vertical farming can make optimal use of space and reduce land usage. At the same time, automated monitoring systems can track the insects’ growing environment in real time, increasing farming efficiency and lowering costs [267,268]. Progress in feed formulation and insect genetics will also further enhance production efficiency. By optimizing feed formulations to provide insects with more suitable nutrition and combining genetic improvement techniques, such as selective breeding and genetic modification, it will be possible to cultivate insect species with better growth performance and higher protein content [269].

In terms of product development and application areas, the potential applications of insect protein are vast. Several insect species have already been found to possess unique value in functional foods and pharmaceuticals. For example, silkworm larvae are used in studies on protein solubility, oil absorption, and foam stability [270]; yellow mealworm larvae are researched for their oil, foam, and emulsifying capabilities [100]; black soldier flies contain antibacterial peptides that target Helicobacter pylori [271], the bacteria responsible for stomach ulcers; and male silkworm pupae extracts are used to treat erectile dysfunction [272]. Future efforts should continue to explore the potential of insects in these areas and develop more products with specific functions. For instance, insect protein could be used to develop more medications or nutritional supplements targeted at specific diseases to meet people’s increasingly diverse health needs. In the feed industry, insect protein-based feeds should be developed based on the nutritional needs of different farmed animals to improve farming efficiency.

Moreover, consumer acceptance is an important factor influencing the promotion of insect protein. Western consumers generally resist consuming insects, with food neophobia and disgust being the main barriers [272,273]. In the future, it will be necessary to change consumer perceptions through educational campaigns and marketing activities, increasing public awareness of the benefits of insect protein. Emphasizing the high nutritional value, environmental friendliness, and unique functional properties of insect protein while improving the taste, texture, and presentation of insect-based foods will enhance their sensory appeal [274]. For example, developing tastier insect-based foods will allow consumers to accept insect protein as they gradually enjoy delicious meals.

With the growing global population, increasing protein demand, and heightened attention to sustainable development, insect protein, as a sustainable and nutritious alternative protein source, is expected to become one of the mainstream sources of protein in the future. Through ongoing efforts in processing technology, production scale, product development, and consumer acceptance, insect protein will be crucial in addressing global challenges such as food security, environmental sustainability, and public health [12,274]. Cross-disciplinary collaboration and joint efforts will be essential to realizing this vision and fully unlocking the potential of insect protein.

## Data Availability

Data sharing is not applicable to this article as no new data were created or analyzed in this study.
